# STK38-mediated feedback loop regulation of the hedgehog pathway governing tumor heterogeneity in renal papillary carcinoma

**DOI:** 10.1038/s41419-025-08225-4

**Published:** 2026-01-15

**Authors:** Yifan Du, Xiuyuan Sui, Zeyuan Zheng, Zhengying Zhang, Bin Liu, Yang Bai, Yue Zhao, Qingqing Wu, Haodong Wu, Min Zhong, Liyan Li, Huimin Sun, Chen Shao

**Affiliations:** 1 Department of Urology, Xiang’an Hospital of Xiamen University, School of Medicine, Xiamen University, Xiamen, China; 2 Central Laboratory, School of Medicine, Xiamen University, Xiamen, China; 3 Rappaport-Technion Integrated Cancer Center, The Rappaport Faculty of Medicine and Research Institute, Technion-Israel Institute of Technology, Haifa, Israel

**Keywords:** Renal cell carcinoma, Cancer stem cells, Cell signalling

## Abstract

Papillary renal cell carcinoma (pRCC) is characterized by marked intratumoral heterogeneity, which contributes to therapeutic resistance and disease progression. In this study, we identify STK38 as a key regulator of tumor heterogeneity in pRCC, functioning through non-canonical activation of the Hedgehog (Hh) signaling pathway. STK38 interacts with both KIF7 and GSK3β to promote Hh signaling by facilitating KIF7 ciliary localization and reprogramming GSK3β substrate selectivity, leading to GLI1 stabilization and β-catenin suppression. Moreover, GLI1 directly enhances *STK38* transcription, establishing a positive feedback loop that reinforces pathway activation. Notably, depletion of STK38 sensitizes tumor cells to a NETosis-like chromatin release process (tNET release), a form of stress-induced nuclear expulsion associated with immune evasion and metastatic potential. Given the potential pro-metastatic consequences of STK38 inhibition, we instead targeted its downstream effector GLI1 using Glabrescione B, which potently suppressed tumor growth and induced apoptosis in both xenograft and patient-derived organoid models, particularly in STK38-high tumors. These findings position STK38 as a critical modulator of pRCC heterogeneity and support GLI1 inhibition as a promising strategy to disrupt oncogenic signaling while minimizing adverse effects.

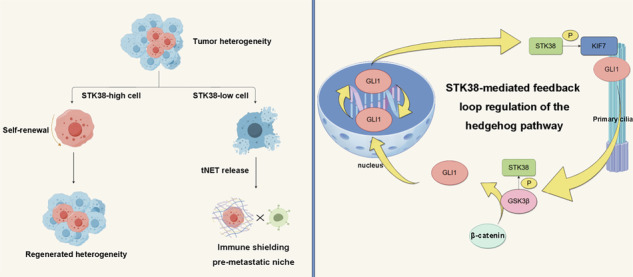

## Introduction

Tumor heterogeneity reflects the diversity among cancer cell populations and can be broadly categorized into inter-tumoral and intratumoral heterogeneity [1]. While the former refers to variations across tumors from different patients, the latter highlights cellular diversity within a single tumor. Intratumoral heterogeneity is a major driver of differential treatment responses and therapeutic resistance, largely attributed to the presence of tumor cell subpopulations with self-renewal and multilineage differentiation capacity. These stem-like cells, akin to normal stem cells, can sustain tumor propagation and contribute to relapse. Under selective pressure from the microenvironment or therapy, more differentiated cancer cells may dedifferentiate into stem-like states, thereby maintaining tumor growth and cellular plasticity [2, 3]. This dynamic regulation is orchestrated by intricate intracellular signaling pathways and microenvironmental cues, both of which play pivotal roles in sustaining regenerative potential. Although numerous surface markers have been proposed to identify such cell states across malignancies, the search for cancer-type-specific indicators remains ongoing and is critical for therapeutic precision.

Papillary renal cell carcinoma (pRCC), a prominent subtype of renal cell carcinoma (RCC), exhibits considerable molecular and histopathological heterogeneity [4, 5]. This complexity contributes significantly to its poor prognosis, particularly in metastatic cases, where therapeutic responses remain inferior to those observed in clear cell RCC (ccRCC) [6, 7]. The conventional histological classification of pRCC into type I and type II has recently been questioned, as growing evidence reveals substantial morphological overlap between the subtypes and no significant prognostic differences after adjustment for TNM staging—suggesting that these classifications may, in fact, represent different stages of the same disease continuum. Despite advances in elucidating the molecular underpinnings of pRCC, current therapeutic approaches—such as MET-targeted tyrosine kinase inhibitors—have achieved only modest clinical benefit. This underscores an urgent need to uncover deeper regulatory mechanisms driving therapeutic resistance and tumor progression. Emerging studies suggest that tumor heterogeneity arises not only from genetic mutations but also from differences in the signaling context of tumor-initiating cells, as well as the spatial and temporal evolution of tumor clones. These dynamic changes profoundly influence tumor biology and treatment response, complicating the clinical management of pRCC. Therefore, a comprehensive understanding of the molecular networks governing tumor progression and intratumoral heterogeneity is essential for the development of more effective and personalized therapeutic strategies.

Among these regulatory networks, the Hedgehog (Hh) signaling pathway has garnered attention due to its critical roles in embryonic development, tissue homeostasis, and cell fate determination [8–10]. Dysregulation of Hh signaling has been implicated in the initiation and progression of multiple cancer types. Canonically, Hh signaling is activated when ligands (e.g., Sonic Hedgehog) bind to the Patched (PTCH) receptor, relieving its inhibition of Smoothened (SMO), which subsequently facilitates the nuclear translocation of GLI transcription factors and activation of downstream genes. Although the core components of the pathway—SMO, GLI1/2, and PTCH—have been extensively studied, less is known about the intermediate regulatory processes, particularly those involving ciliary trafficking and protein phosphorylation. Of particular interest is the primary cilium, a specialized organelle that orchestrates the spatial dynamics of Hh signaling. The kinesin protein KIF7 plays a critical role in modulating the subcellular localization and transcriptional activity of GLI proteins. Aberrations in ciliary function or KIF7 localization can lead to aberrant Hh signaling, promoting tumor aggressiveness and heterogeneity. In pRCC, where MET mutations are frequently observed, these aberrations may synergize with oncogenic signals, contributing to tumor progression.

STK38 (also known as NDR1), a serine/threonine kinase from the AGC kinase family, was initially discovered in yeast as part of the evolutionarily conserved nuclear Dbf2-related (NDR) kinase family. STK38 has been implicated in diverse cellular processes, including apoptosis, immune regulation, inflammation, and cytoskeletal dynamics [11–15]. Despite its broad involvement in various biological pathways, the functional dichotomy of STK38 in different tissues and tumor contexts remains poorly understood. Its potential role in mediating tumor heterogeneity has only recently begun to gain attention. Emerging data suggest that STK38 may act as a central modulator of signaling pathways that govern tumor plasticity and differentiation. For instance, STK38 has been reported to interact with components of the Wnt and Notch pathways—two key signaling axes involved in tumor progression. Given its role in multiple signaling networks, STK38 is poised to serve as a crucial regulator of tumor heterogeneity, with potential implications for therapy resistance and disease relapse.

In this study, we provide evidence that STK38 modulates Hedgehog signaling by interacting with KIF7, enhancing its phosphorylation and promoting its localization to the ciliary tip. Moreover, STK38 interacts with GSK3β, modulating the interplay between Hedgehog and Wnt signaling pathways. Specifically, STK38 suppresses GSK3β phosphorylation, shifting its substrate preference from GLI1 to β-catenin. In turn, activated GLI1 transcriptionally upregulates *STK38*, establishing a feedforward loop that amplifies Hedgehog signaling while simultaneously repressing Wnt activity. This dual regulatory mechanism enhances intratumoral heterogeneity and drives malignant progression in pRCC. Collectively, our findings identify STK38 as a novel mediator of signaling heterogeneity and a promising therapeutic target in papillary renal cell carcinoma.

## Results

### Heterogeneous expression of *STK38* reflects tumor-initiating and self-renewing programs in pRCC

**Fig. 1:**
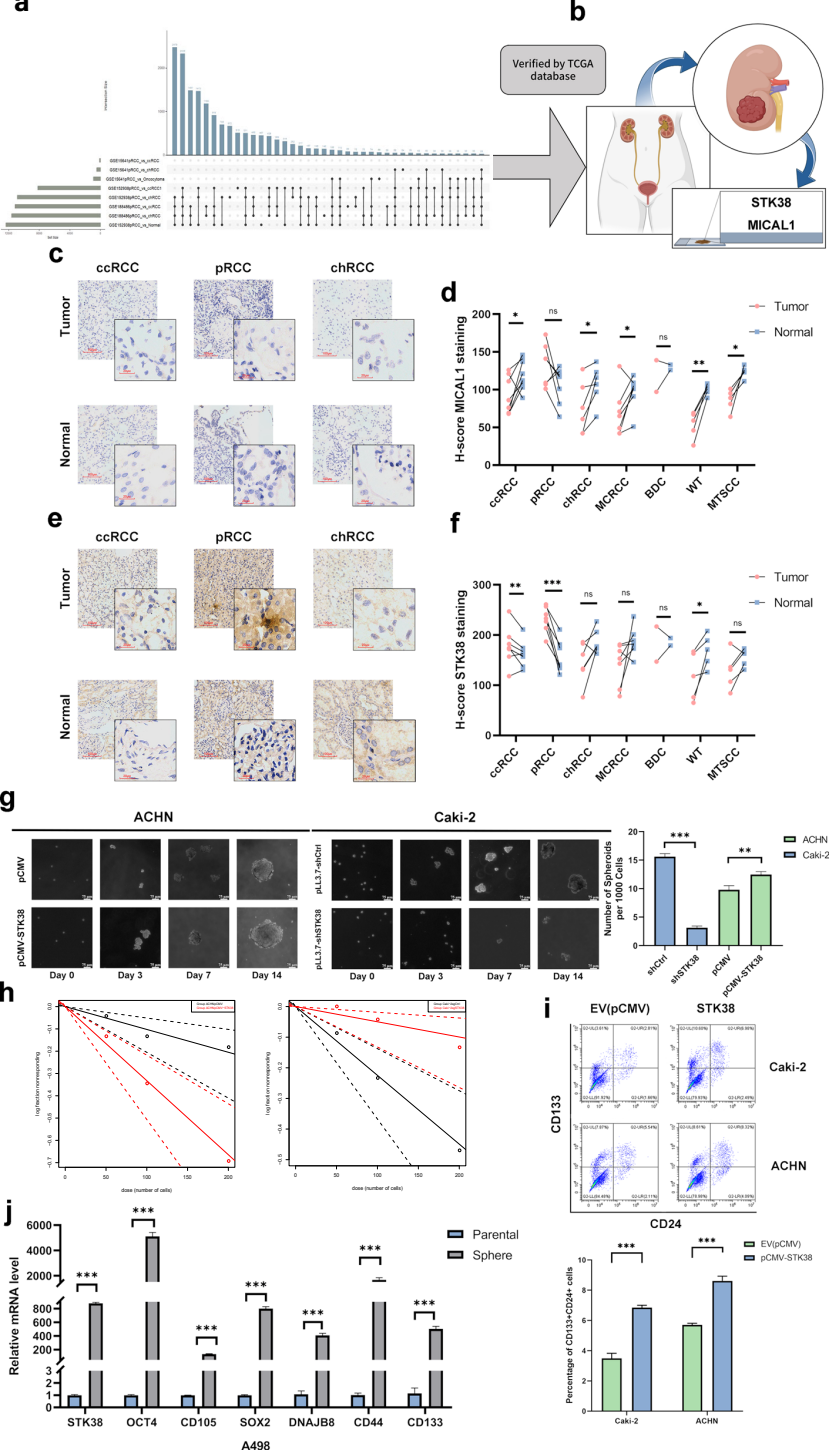
STK38 is selectively upregulated in papillary renal cell carcinoma and drives tumor-initiating and self-renewal programs. **a** Schematic workflow of differential expression analysis. Transcriptomic data from three GEO datasets (GSE15641, GSE152938, and GSE188486), including various renal cancer subtypes and normal tissues, were analyzed to identify genes differentially expressed in pRCC. Genes with adjusted *p* < 0.05 and |log₂FC| > 0.5 were selected. Overlapping candidates were visualized by an UpSet plot, followed by validation using TCGA pRCC cohorts (see Supplementary Fig. S1a–f), leading to the identification of *MICAL1* and *STK38*. **b** Diagram illustrating immunohistochemical (IHC) screening of candidate genes in tissue samples from patients with different renal cancer subtypes. **c** Representative IHC images of MICAL1 expression in tumor and matched adjacent non-tumor tissues from patients with clear cell RCC (ccRCC), papillary RCC (pRCC), and chromophobe RCC (chRCC). Images were acquired at ×20 and ×40 objective magnification. **d** Quantification of MICAL1 IHC staining across tumor and adjacent tissues (n = 40, matched pairs across subtypes). Data shown as mean ± SD. **e** Representative IHC images showing STK38 expression in tumor and adjacent tissues in ccRCC, pRCC, and chRCC. **f** Quantitative analysis of STK38 IHC staining reveals that STK38 expression is markedly elevated in pRCC tumors compared to adjacent tissues and other RCC subtypes. **g** Tumorsphere formation assay performed using *STK38*-overexpressing ACHN cells and *STK38*-knockdown Caki-2 cells, along with corresponding controls. Cells were cultured in serum-free DMEM/F12 or McCoy’s 5A medium supplemented with EGF, bFGF, and N2. Spheres were imaged at days 0, 3, 7, and 14. Spheres >75 µm were counted, and the number of spheres per 1000 seeded cells is shown (right panel). **h** Extreme-limiting dilution assay (ELDA) comparing tumor-initiating frequency in control versus *STK38*-overexpressing or -knockdown cells. **i** Flow cytometry analysis of CSC surface markers CD24 and CD133 in Caki-2 and ACHN cells following STK38 overexpression. The percentage of double-positive CD133+CD24+ cells was quantified. Representative flow cytometry plots (top) and bar graph (bottom) are shown. **j** RT-qPCR analysis of *STK38* and self-renewal-associated genes (e.g., *CD44*, *SOX2*, *OCT4*) in A498 tumorspheres versus adherent cultures. Statistical comparisons were performed using Student’s t-test or one-way ANOVA where appropriate. *P < 0.05, **P < 0.01, ***P < 0.001. Data are presented as mean ± SD of three independent experiments.

To identify molecular drivers of intratumoral heterogeneity in papillary renal cell carcinoma (pRCC), we performed integrated differential expression analyses using three independent GEO datasets (GSE15641, GSE152938, GSE188486), encompassing various renal cancer subtypes and normal tissues (Fig. 1a). Genes meeting stringent criteria (adjusted *p* < 0.05, |log₂FC| > 0.5) and consistently identified across all datasets were further validated using the TCGA-KIRP cohort, resulting in two robust candidate genes: *MICAL1* and *STK38* (Figs. 1b and S1a–f). Subsequent immunohistochemical (IHC) analysis of tissue samples from a diverse clinical cohort—including both common renal cancer subtypes (pRCC, ccRCC, chRCC) and rarer malignancies (e.g., multilocular cystic RCC, Bellini duct carcinoma, Wilms tumor, and mucinous tubular and spindle cell carcinoma)—revealed that STK38 was most prominently and specifically overexpressed in pRCC (Fig. 1c–f). This broad inclusion of renal cancer subtypes was essential for capturing the full spectrum of molecular variation across these tumors, enabling us to identify genes consistently dysregulated in pRCC while distinguishing them from other renal malignancies. These comprehensive screening and validation results underscore STK38 as a compelling candidate for further mechanistic exploration.

Functional enrichment analysis of *STK38*-associated transcriptional signatures in pRCC (Fig. S1g) revealed strong associations with gene programs related to tumor initiation and self-renewal, implicating STK38 as a potential regulator of intratumoral heterogeneity. To functionally validate this, we manipulated STK38 expression in a panel of renal carcinoma cell lines with documented relevance to papillary RCC biology [16–18]: Specifically, STK38 was overexpressed in ACHN cells, which exhibit type I pRCC-like features and low endogenous STK38 expression; knocked down in Caki-2 cells, which have been increasingly recognized as molecular surrogates of type II pRCC and express high levels of STK38; and further validated in A498 cells, considered a transcriptionally intermediate subtype bridging clear cell and papillary features. These three models were selected to reflect a functional continuum of STK38 expression and pRCC subtype heterogeneity, thereby enhancing the robustness of pathway interrogation. In 3D tumorsphere formation assays, STK38 overexpression in ACHN significantly enhanced both sphere formation and growth, while knockdown in Caki-2 markedly impaired these phenotypes (Fig. 1g).

Extreme-limiting dilution assays further quantified the frequency of tumor-initiating cells, confirming that *STK38* overexpression significantly enhanced tumorigenic capacity in vitro (Fig. 1h). The detailed limiting dilution data are provided in Supplementary Table 2. Consistently, flow cytometric analysis using established surface markers CD24 and CD133 revealed that STK38 upregulation significantly increased the proportion of CD133+CD24+ double-positive cells in Caki-2 and ACHN cells, supporting its role in sustaining self-renewing tumor phenotypes (Fig. 1i).

To explore the in vivo relevance of these findings, we analyzed single-cell RNA sequencing data from pRCC patients (GSE152938) using Seurat-based unsupervised clustering, which identified 12 transcriptionally distinct cellular clusters (Fig. S1h). Although *STK38* expression was not confined to a single dominant cluster, it was detectable across multiple tumor-associated clusters. Feature plot analysis further revealed that *STK38* partially overlapped with stemness-associated markers (e.g., *CD44*, *ALDH1A1*, *NANOG*), whose expression was also broadly distributed across clusters (Fig. S1i). Additionally, transcriptomic profiling of pRCC cells cultured under sphere-forming versus monolayer conditions showed consistent upregulation of stemness-related genes in STK38-overexpressing cells (Fig. 1j), further linking STK38 to the maintenance of undifferentiated tumor subpopulations. These findings suggest that while STK38 is not a canonical stemness marker, its expression is enriched in transcriptional neighborhoods associated with regenerative gene programs, consistent with its proposed role in supporting tumor plasticity.

### STK38 drives tumor initiation and self-renewal through hedgehog pathway activation

**Fig. 2:**
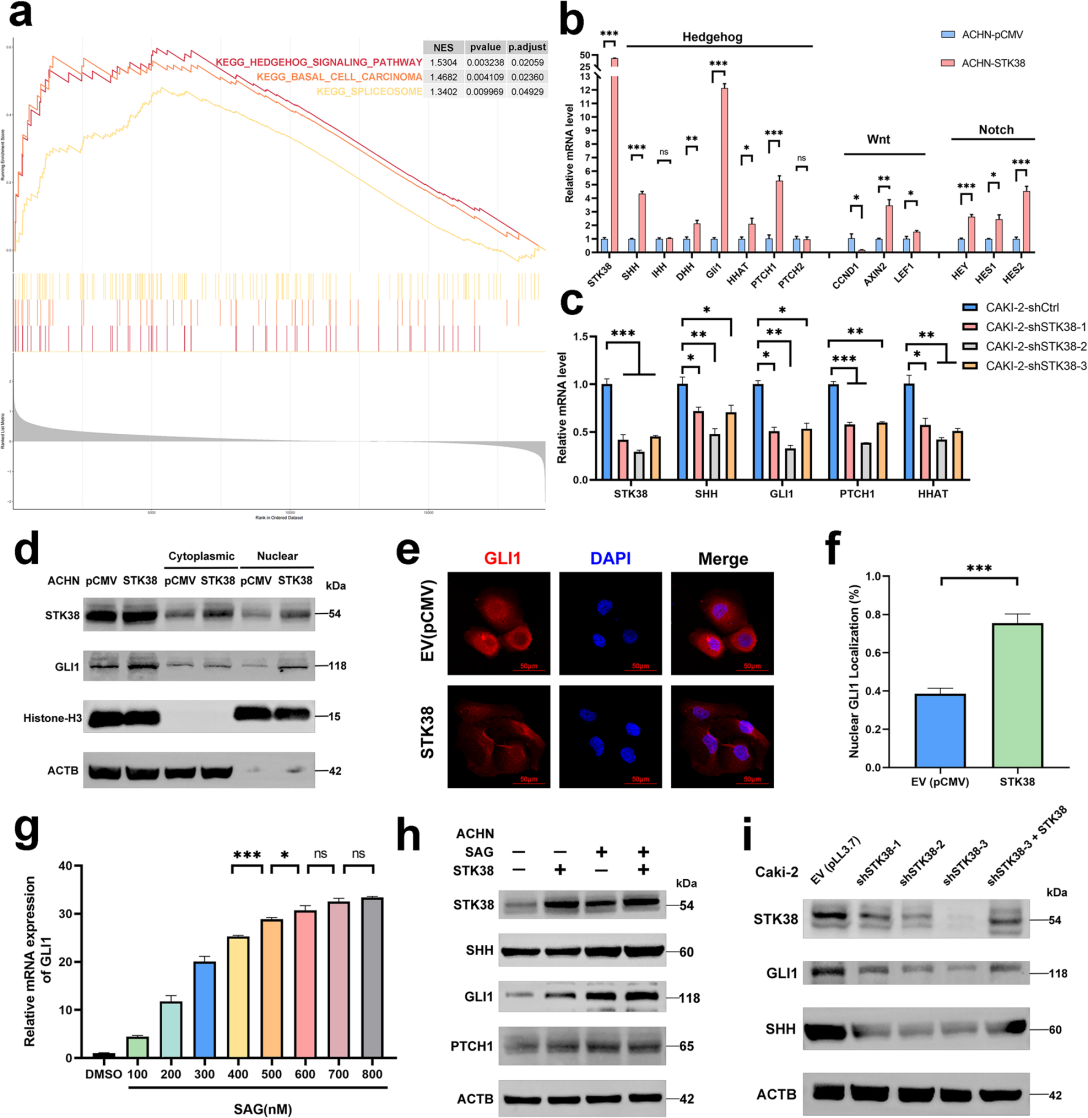
STK38 activates Hedgehog signaling through a non-canonical mechanism to promote tumor initiation in pRCC. **a** Gene set enrichment analysis (GSEA) was performed using TCGA transcriptomic data from pRCC patients. Samples were stratified into top and bottom 40% based on *STK38* expression, and differentially expressed genes were analyzed. Hh signaling emerged as the most significantly enriched pathway. **b** RT-qPCR analysis of downstream targets from Hedgehog, Wnt, and Notch pathways in ACHN cells overexpressing *STK38*. *STK38* overexpression preferentially upregulated Hh pathway targets. **c** RT-qPCR showing the expression of Hh downstream targets following *STK38* knockdown using three independent shRNA constructs in ACHN cells, confirming *STK38*-dependent regulation of the Hh pathway. **d** Western blot analysis of GLI1 localization in nuclear and cytoplasmic fractions from ACHN cells with or without STK38 overexpression. **e** Immunofluorescence staining of GLI1 in control and STK38-overexpressing ACHN cells revealed enhanced nuclear accumulation of GLI1 upon STK38 overexpression. **f** Quantification of GLI1 nuclear localization based on fluorescence intensity using ImageJ analysis. Data represent mean nuclear fluorescence per cell across multiple fields (n > 50 cells per group). **g** Determination of optimal SAG (Smoothened agonist) saturation concentration by measuring GLI1 mRNA levels across a gradient of SAG concentrations; pathway activation plateaued at 700 nM. **h** Western blot analysis of GLI1 expression in ACHN cells treated with or without SAG (600 nM) and with or without STK38 overexpression. Persistent GLI1 upregulation under SAG-saturated conditions indicates STK38 activates Hh signaling via a non-canonical pathway. **i** Rescue experiment demonstrating the specificity of STK38-mediated Hh activation. STK38 was silenced using three distinct shRNA constructs, and Hh target gene expression was assessed. Reintroduction of STK38 in sh-STK38-3 cells restored Hh target gene expression levels. Data are presented as mean ± SD from three independent experiments. *P < 0.05, **P < 0.01, ***P < 0.001. Statistical significance was determined using Student’s t-test or one-way ANOVA.

To investigate the signaling mechanisms through which STK38 promotes tumor initiation and self-renewal capacity, gene set enrichment analysis (GSEA) was conducted using transcriptomic profiles from pRCC patient cohorts. STK38 expression was significantly enriched in multiple intracellular signaling programs, with the Hedgehog (Hh) pathway showing the strongest positive correlation (Fig. 2a).

Given the well-established role of Hh signaling in supporting tumor-initiating potential across cancers, we next validated its relevance in pRCC cell lines. Comparative analysis of three canonical developmental pathways—Hedgehog, Wnt, and Notch—revealed that STK38 most robustly modulated the Hh cascade, consistent with the GSEA findings (Fig. 2b, c). Since GLI1 nuclear translocation is a hallmark of Hedgehog pathway activation, and this pathway is known to auto-regulate, we conducted nuclear–cytoplasmic fractionation assays (Fig. 2d), as well as immunofluorescence analyses (Fig. 2e), both of which confirmed that STK38 overexpression enhances GLI1 nuclear localization (Fig. 2f), indicating activation of Hedgehog signaling.

To determine whether STK38 activates the Hedgehog pathway through canonical or non-canonical mechanisms, we employed Smoothened agonist (SAG) to saturate the PTCH receptor (Fig. 2g). Notably, STK38 continued to promote Hedgehog signaling even under PTCH-saturated conditions (Fig. 2h), suggesting that it acts via a non-canonical route, independent of upstream receptor activation.

Finally, to validate the specificity of STK38-mediated Hedgehog activation, we reintroduced *STK38* into cells silenced by the most effective shRNA (sh-STK38-3). This re-expression successfully restored downstream signaling, confirming the functional relevance of STK38 in Hedgehog pathway regulation (Fig. 2i).

### STK38 selectively activates the Hedgehog pathway via interactions with KIF7 and GSK3β

**Fig. 3:**
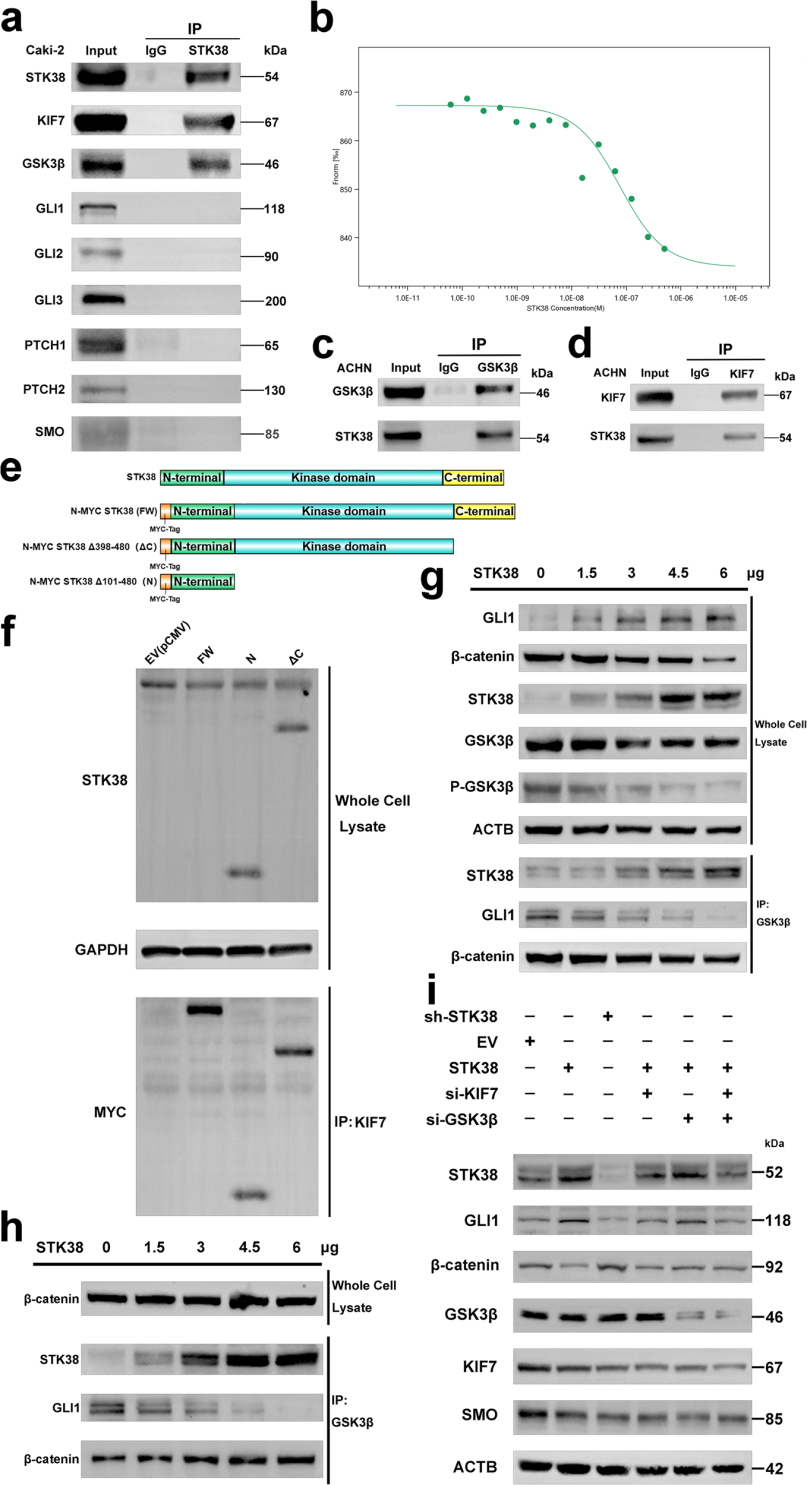
STK38 selectively activates Hedgehog signaling via interaction with KIF7 and reprogramming of GSK3β substrate preference. **a** Co-immunoprecipitation (Co-IP) assay showing interaction between endogenous STK38 and GSK3β in Caki-2 cells. **b** Microscale thermophoresis (MST) analysis of STK38–KIF7 binding. Fluorescently labeled KIF7 was held at a fixed concentration while STK38 was serially diluted (1:2 across 18 capillaries). The thermophoretic signal decreased with reduced STK38 concentration and plateaued at low levels, indicating a concentration-dependent binding interaction. **c**, **d** Reciprocal Co-IP assays in ACHN cells confirming endogenous STK38–KIF7 and STK38–GSK3β interactions. **e** Schematic representation of STK38 truncation mutants used for mapping KIF7 interaction domains. **f** Co-IP assays using endogenous KIF7 to pull down Myc-tagged STK38 truncation constructs expressed in ACHN cells. STK38 truncations were designed with N-terminal Myc tags, allowing immunodetection of each construct via anti-Myc following immunoprecipitation with KIF7. Notably, since the STK38 antibody used for immunoblotting recognizes an N-terminal epitope, it was also able to detect truncated constructs retaining this region, leading to visible bands even in the non-Myc detection channel. Results revealed that both the N-terminal and kinase domains of STK38 contribute to KIF7 binding, while deletion of the C-terminal region had minimal effect. **g** Western blot analysis of GLI1 and β-catenin expression following graded overexpression of STK38 in ACHN cells; total levels and co-IPs from the same lysates are shown. **h** After normalizing β-catenin expression across conditions, GSK3β co-IP indicates a shift in substrate association—reduced binding to GLI1 and increased binding to β-catenin upon STK38 overexpression. **i** Rescue experiment validating that STK38 activates Hedgehog signaling through both KIF7 and GSK3β. In ACHN cells, STK38 was overexpressed alone or in combination with siRNAs targeting KIF7 and/or GSK3β. Hh pathway target expression was assessed by qRT-PCR. Combined knockdown attenuated STK38-induced pathway activation, confirming dual effector dependence. Data are shown as mean ± SD from three independent experiments. *P < 0.05, **P < 0.01, ***P < 0.001. Statistical significance was determined by Student’s t-test or one-way ANOVA.

To dissect the mechanism by which STK38 activates the Hedgehog (Hh) pathway, we performed co-immunoprecipitation (co-IP) followed by liquid chromatography–tandem mass spectrometry (IP–MS), which identified KIF7 and GSK3β among STK38-associated proteins; their association was validated by co-IP (Fig. 3a, c, d; Supplementary Table 3). Candidates were prioritized by intersecting the IP–MS list with the Hedgehog pathway. To further characterize the interaction between STK38 and KIF7, we employed microscale thermophoresis (MST) using the NanoTemper Monolith Pico system. Fluorescently labeled KIF7 was held at a constant concentration while STK38 was titrated across a concentration gradient. The binding affinity was quantified by monitoring the thermophoretic signal, which decreased progressively with lower STK38 concentrations and plateaued at low-dose ranges, indicating a specific and concentration-dependent interaction between STK38 and KIF7 (Fig. 3b).

To map the STK38 domains required for this interaction, we generated truncation mutants. Deletion of the C-terminal region did not abrogate KIF7 binding (Fig. 3e, f), consistent with our molecular docking simulations, which identified key interaction residues (PHE-280, LEU-27, MET-22, VAL-114, ASP-110, and LYS-109) within the N-terminal and kinase domains (Fig. S2f). Given that KIF7 is essential for Hedgehog signaling via its ciliary tip localization, we investigated whether STK38 influences this process. Our data showed that STK38 promotes KIF7 phosphorylation (Fig. S2c) and enhances its accumulation at the ciliary tip (Fig. S2a, b), thereby facilitating Hh pathway activation.

Interestingly, STK38 also interacts with GSK3β, a well-characterized tumor suppressor and serine/threonine kinase that promotes substrate degradation via phosphorylation. Prior studies have shown that GSK3β-mediated phosphorylation of GLI1 and β-catenin leads to their degradation through the β-TrCP-mediated ubiquitin-proteasome pathway. Given that STK38 has previously been reported to inhibit β-catenin activity in prostate cancer, we hypothesized that STK38 might modulate GSK3β substrate preference in pRCC.

To test this, we examined GSK3β binding to GLI1 and β-catenin under increasing levels of STK38 expression. As STK38 levels increased, GSK3β phosphorylation was reduced, and its binding affinity to GLI1 decreased, whereas its interaction with β-catenin remained largely unchanged (Fig. 3g). Since this may be confounded by overall β-catenin protein levels, we adjusted β-catenin expression to comparable levels and repeated the immunoprecipitation assay. Under these conditions, STK38 enhanced GSK3β binding to β-catenin while reducing its affinity for GLI1, suggesting that STK38 selectively shifts GSK3β substrate preference, thereby stabilizing GLI1 and activating Hedgehog signaling (Fig. 3h).

To functionally validate that STK38 mediates Hedgehog pathway activation through both KIF7 and GSK3β, we performed rescue experiments by overexpressing STK38 in pRCC cells, followed by siRNA-mediated knockdown of KIF7, GSK3β, or both. The knockdown efficiency of each siRNA was confirmed by immunoblotting, and the most efficient constructs were selected for further analysis (Fig. S2d, e). Notably, silencing KIF7 markedly attenuated STK38-induced Hedgehog activation, whereas GSK3β knockdown alone paradoxically enhanced pathway activity, consistent with its known role as a negative regulator of GLI1 stability. However, when both KIF7 and GSK3β were silenced in the context of STK38 overexpression, Hedgehog pathway activity was significantly diminished compared to STK38 overexpression alone, indicating that STK38 promotes Hedgehog signaling via a dual mechanism: enhancing KIF7 function while reprogramming GSK3β substrate selectivity (Fig. 3i).

### GLI1 transcriptionally activates *STK38*, establishing a positive feedback loop in pRCC

**Fig. 4:**
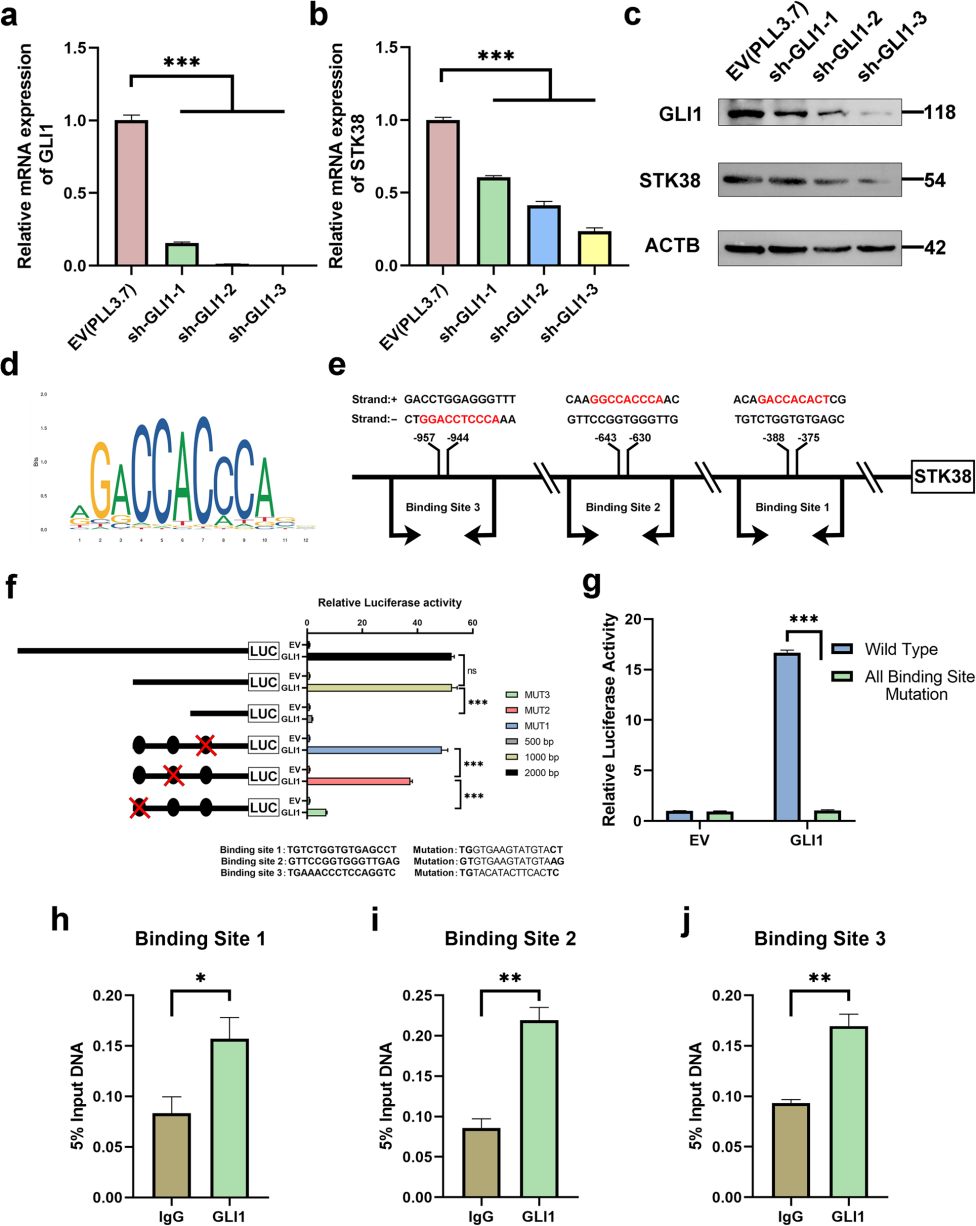
GLI1 directly binds the STK38 promoter and establishes a positive feedback loop that amplifies Hedgehog signaling. **a** *GLI1* knockdown efficiency was validated in Caki-2 cells using three independent shRNA constructs. *GLI1* mRNA levels were measured by qRT-PCR. **b** *STK38* mRNA expression decreased following *GLI1* silencing, indicating potential transcriptional regulation. **c** Western blot confirming that GLI1 knockdown also reduced STK38 protein levels in Caki-2 cells. **d** Predicted GLI1-binding motifs within the *STK38* promoter region (−2000 to 0 bp upstream of the transcription start site), identified using JASPAR and UCSC Genome Browser databases. **e** Schematic representation of three putative GLI1-binding sites in the *STK38* promoter. **f** Dual-luciferase reporter assays with a series of promoter truncations (−2000 bp, −1000 bp, and −500 bp) revealed that the GLI1-responsive region is located between −1000 and −500 bp. Site-directed mutagenesis of the three predicted binding sites showed that site 1 had no measurable effect, site 2 had a moderate effect, and site 3 was the primary contributor to GLI1-mediated transcriptional activation. **g** A luciferase reporter carrying all three binding-site mutations exhibited a substantial reduction in activity compared to the wild-type promoter, confirming the absence of additional unidentified GLI1-responsive elements. **h**–**j** ChIP-qPCR assays with anti-GLI1 antibody validated direct binding of GLI1 to sites 1, 2, and 3. Strongest enrichment was observed at site 3, followed by site 2, while site 1 showed minimal occupancy. Data are presented as mean ± SD from three independent experiments. *P < 0.05, **P < 0.01, ***P < 0.001. Statistical significance was determined using Student’s t-test or one-way ANOVA.

Interestingly, during the rescue experiments described above, we observed that manipulation of downstream Hedgehog pathway components also impacted STK38 expression itself. Specifically, suppression of GLI1, the terminal effector of Hedgehog signaling, led to a marked reduction in both *STK38* mRNA (Fig. 4a, b) and protein levels (Fig. 4c). These findings raised the possibility that STK38 is not only an upstream modulator but also a downstream transcriptional target of Hedgehog signaling, potentially forming a positive feedback loop.

To explore this hypothesis, we first analyzed publicly available datasets, which revealed a significant positive correlation between *GLI1* and *STK38* expression in pRCC (Fig. S3a). Using the UCSC Genome Browser in conjunction with JASPAR binding motif predictions, we identified three putative GLI1-binding sites within the 2 kb upstream promoter region of *STK38* (Fig. 4d, e). Dual-luciferase reporter assays with progressive promoter truncations indicated that the −1000 to −500 bp region upstream of the transcription start site harbors major GLI1-responsive elements (Fig. 4f). Site-directed mutagenesis of individual predicted binding sites, along with comparison between wild-type and fully mutated promoter constructs, demonstrated that site 2 and site 3 are critical for GLI1-driven transactivation (Fig. 4g).

To further confirm direct binding, we performed ChIP-qPCR using primers specifically targeting the predicted sites. Enrichment of GLI1 at these regions, particularly site 3, was clearly detected (Fig. 4h–j), confirming that GLI1 directly binds to the *STK38* promoter and activates its transcription.

Together, these findings uncover a previously uncharacterized positive feedback loop in which STK38 promotes Hedgehog pathway activation via KIF7 and GSK3β, while GLI1 transcriptionally reinforces *STK38* expression. This signaling circuit amplifies Hedgehog output and may sustain self-renewing tumor subpopulations in pRCC.

### Low STK38 expression in tumor cells facilitates NETosis-like chromatin release

During our tumorsphere formation assays, we observed that *STK38*-knockdown pRCC cells exhibited unique morphological changes not typical of apoptosis. Notably, these cells were surrounded by reticular, debris-like structures resembling Neutrophil Extracellular Traps (NETs) (Fig. S3b). This observation suggests that these tumor cells might undergo a process akin to NETosis, termed tumor cell NETosis-like Chromatin Release (tNET release), wherein chromatin is expelled extracellularly, potentially modifying the tumor microenvironment and promoting immune evasion and metastasis.

**Fig. 5:**
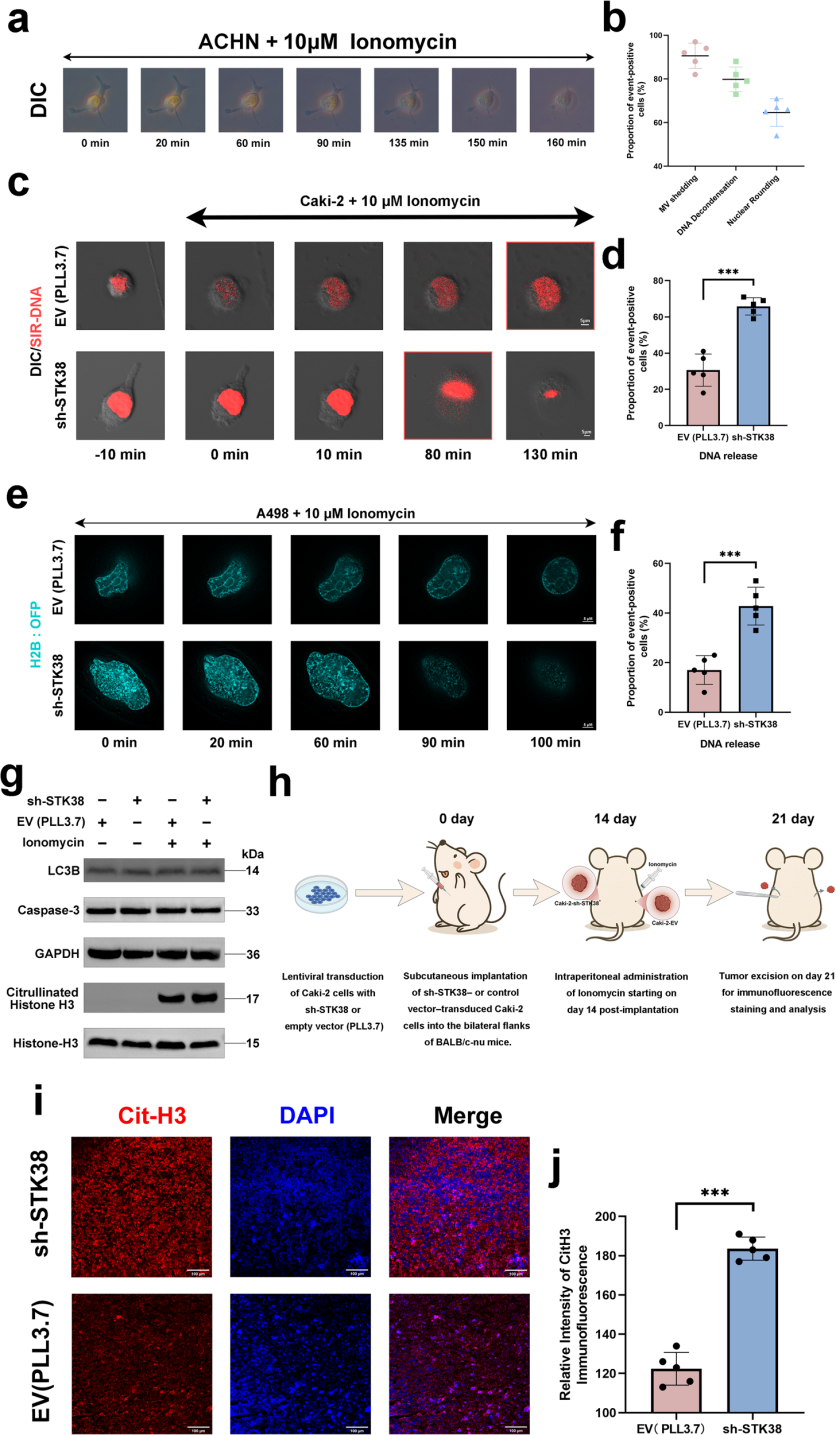
Loss of STK38 sensitizes tumor cells to NETosis-like chromatin release under stress conditions. **a** Ionomycin treatment (10 μM, 160 min) induces chromatin release in ACHN cells, characterized by DNA decondensation, membrane blebbing, and nuclear rounding, as observed by DIC microscopy. **b** Quantification of key morphological stages during tNET release, including DNA decondensation, membrane vesicle (MV) shedding, and nuclear rounding. Data represent the percentage of affected cells from a total of 203 cells, averaged across five independent replicates. **c** Live-cell imaging using SiR-DNA staining in control and *STK38*-knockdown Caki-2 cells. Time-lapse images were acquired using Zeiss LSM 880 confocal microscopy. *STK38* knockdown increased both the frequency and rate of chromatin extrusion. **d** Quantification of the percentage of DNA release-positive cells over a 150-min time course based on SiR-DNA signal dispersion. **e** High-resolution imaging of chromatin release using structured illumination microscopy (SIM) in H2B-OFP-expressing A498 cells. **f** Quantification of H2B-OFP-labeled chromatin release over a 120-min period in control versus *STK38*-knockdown cells. A total of 203 cells were analyzed across five independent experiments. **g** Western blot analysis of citrullinated histone H3 (Cit-H3), LC3B, and cleaved caspase-3 in control and *STK38*-knockdown cells with or without Ionomycin treatment. Enhanced Cit-H3 expression was observed specifically in the *STK38*-depleted group, whereas apoptotic and autophagic markers showed no significant change. **h** Schematic of the in vivo chromatin release experiment. Control and sh-STK38-stable ACHN cells were subcutaneously injected into BALB/c-nu mice. Once palpable tumors formed, mice received intraperitoneal Ionomycin injections for 7 consecutive days prior to tissue harvest. **i** Representative immunofluorescence of H3-Cit (red) and DAPI (blue) in tumors from control and STK38-knockdown groups (scale bar, 100 μm). **j** Quantification of Cit-H3 fluorescence. Each dot represents one tumor (mean of five non-overlapping fields per tumor); values are mean ± SD, n = 5 tumors per group. Data are shown as mean ± SD. *P < 0.05, **P < 0.01, ***P < 0.001. Statistical significance was assessed using Student’s t-test or ANOVA.

To investigate this phenomenon, we first assessed whether pRCC cells could undergo tNET release upon stimulation. Treatment with Ionomycin, a known NETosis inducer, led to observable changes under differential interference contrast (DIC) microscopy, including membrane blebbing, chromatin decondensation, and nuclear rounding—hallmarks of chromatin release (Fig. 5a, b). We hypothesized that STK38 downregulation sensitizes tumor cells to tNET release under stress conditions. To test this, we labeled DNA using SiR-DNA and H2B:OFP, then induced stress with Ionomycin. Live-cell imaging revealed that *STK38*-knockdown cells exhibited accelerated chromatin release and a higher frequency of this event compared to controls (Fig. 5c–f). Western blot analysis further demonstrated increased levels of citrullinated histone H3 (H3-Cit), a NETosis marker, in STK38-deficient cells, distinguishing this process from autophagy and apoptosis (Fig. 5g).

To validate these findings in vivo, we generated xenograft models by implanting sh-STK38- or control vector-transduced Caki-2 cells into BALB/c-nu mice (Fig. 5h). Upon Ionomycin treatment, tumors were excised and subjected to immunofluorescence analysis, which revealed a marked increase in Cit-H3-positive regions in sh-STK38 tumors compared with controls (Fig. 5i). Quantitative assessment confirmed significantly elevated Cit-H3 fluorescence intensity in the STK38-knockdown group, indicating enhanced chromatin release in the absence of STK38 (Fig. 5j).

Collectively, these results indicate that low STK38 expression predisposes tumor cells to undergo tNET release under stress, potentially contributing to immune evasion and metastatic progression.

### Pharmacological inhibition of GLI1 suppresses tumor growth in STK38-high pRCC and represents a promising therapeutic strategy

Given that STK38 activates Hedgehog signaling via non-canonical mechanisms and participates in a positive feedback loop through GLI1, direct inhibition of GLI1 may represent a more effective therapeutic approach than upstream inhibition. Moreover, since STK38 suppression promotes tNET release, which may enhance immune evasion and metastasis, indirect targeting of the STK38-Hedgehog axis also requires careful therapeutic consideration. As SMO inhibition is known to induce compensatory resistance mechanisms, we instead evaluated Glabrescione B (GlaB), a small-molecule GLI1 inhibitor, for its therapeutic potential in pRCC.

**Fig. 6:**
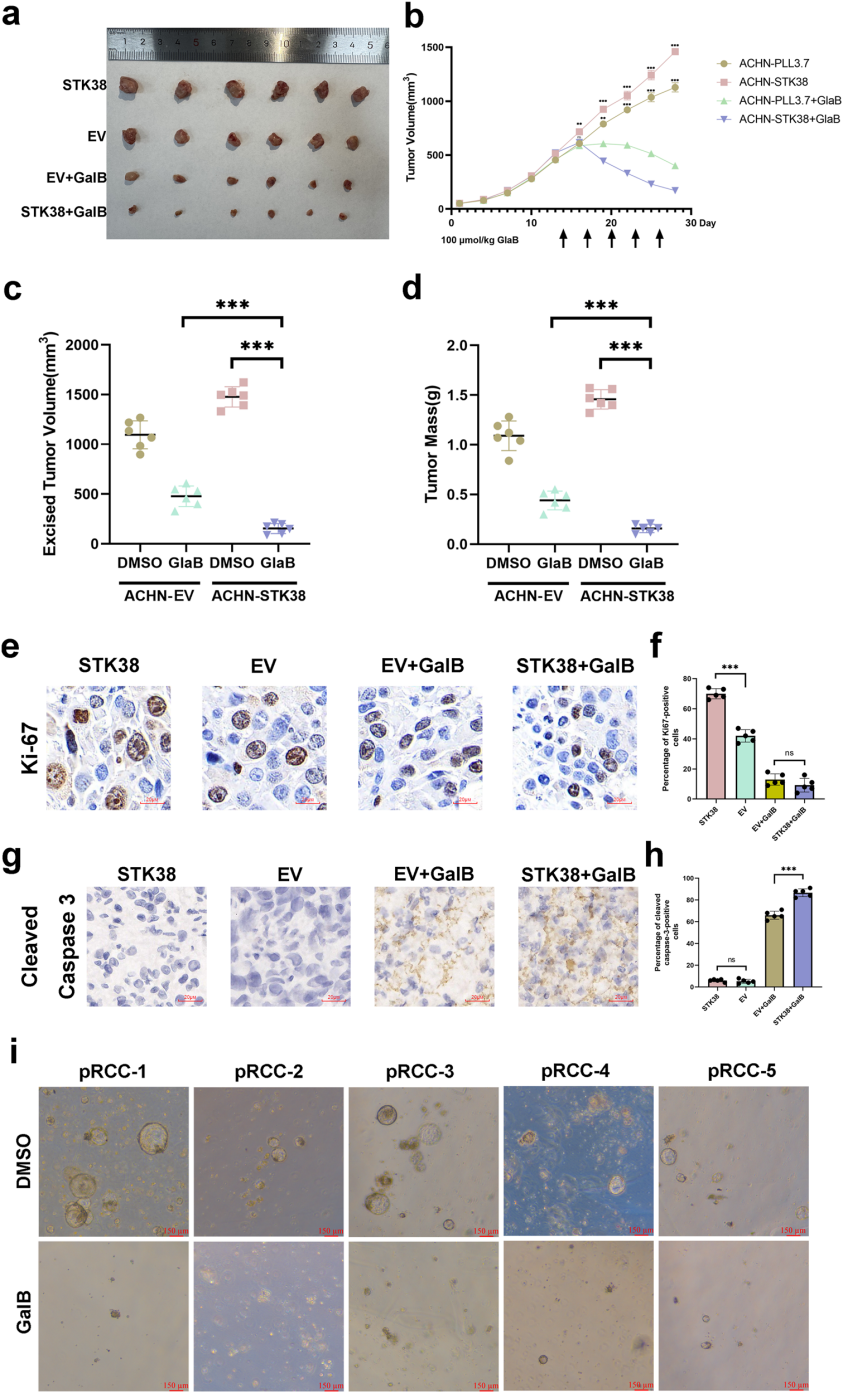
Pharmacological inhibition of GLI1 with Glabrescione B suppresses tumor growth, particularly in STK38-high pRCC models. **a** Representative images of xenograft tumors from four groups: control, *STK38* overexpression, control + Glabrescione B (GlaB), and *STK38* overexpression + GlaB. Each group included six mice. GlaB was administered intraperitoneally every 3 days starting on day 14 post-injection, for a total of five doses. **b** Tumor volumes were measured every 3 days using the formula: volume = (length × width^2^)/2. STK38 overexpression significantly accelerated tumor growth, while GlaB treatment attenuated this effect. Significance stars indicate comparisons between ±GlaB within the same genetic background (i.e., ACHN-pCMV vs. ACHN-pCMV+GlaB; ACHN-STK38 vs. ACHN-STK38+GlaB). **c** Final tumor volumes at the endpoint showed significant differences between overexpression and control groups, as well as between treated and untreated groups. **d** Tumor mass (grams) measured at sacrifice confirmed the growth trends observed in volumetric analysis. **e** Representative Ki67 immunohistochemistry images from all four treatment groups. **f** Quantification of Ki67-positive nuclei per field across five random fields per tumor (n = 6 tumors per group). *STK38* overexpression increased proliferative index, which was reversed by GlaB treatment. **g** Representative immunohistochemistry of cleaved caspase-3 (cl-CASP3) showing enhanced apoptotic signal in GlaB-treated groups. **h** Quantification of cleaved caspase-3-positive cells in tumor sections, calculated as mean number of positive cells per field (n = 6 tumors per group). **i** Ex vivo drug testing in five patient-derived pRCC organoid lines. Organoids were passaged and treated with GlaB or 0.05% DMSO (control) for 7 days. GlaB treatment consistently inhibited organoid expansion across all patient samples. Data are presented as mean ± SD. *P < 0.05, **P < 0.01, ***P < 0.001. Statistical analyses were performed using two-way ANOVA or unpaired t-test as appropriate.

In a xenograft model comprising four experimental groups (control, STK38-overexpression, control + GlaB, and STK38-overexpression + GlaB), GlaB treatment was initiated at week 14 (Fig. 6a). *STK38* overexpression significantly promoted tumor growth, as expected, while GlaB treatment markedly suppressed tumor progression, with the most substantial reduction observed in the *STK38*-overexpressing group treated with GlaB (Fig. 6b–d). These results suggest that pRCC tumors with high *STK38* expression are particularly sensitive to GLI1-targeted therapy.

Immunohistochemical analysis of xenograft tumors revealed increased Ki67 staining in the STK38-overexpressing group, indicating enhanced proliferative activity. Conversely, cleaved Caspase-3 expression was significantly elevated in the STK38 + GlaB group, suggesting that GLI1 inhibition not only halts proliferation but also induces apoptosis in STK38-driven tumors (Fig. 6e–h). Importantly, H&E staining of major organs (heart, liver, spleen, lung, and kidney) revealed no significant histopathological abnormalities, indicating favorable toxicity profiles of GlaB in vivo (Fig. S3c).

To further assess clinical relevance, we tested GlaB in patient-derived organoid (PDO) models from five independent pRCC cases. Organoids were treated on day 7, and untreated controls received 0.05% DMSO. GlaB treatment consistently reduced organoid size and viability, demonstrating potent anti-tumor efficacy in a clinically relevant ex vivo model (Fig. 6i).

Collectively, these findings delineate a dual role for STK38 in pRCC—facilitating Hedgehog pathway-driven tumor initiation and cellular plasticity, while its downregulation predisposes tumor cells to NETosis-like chromatin release, potentially enhancing metastatic dissemination. Based on this, we prioritized targeting the downstream effector GLI1 using Glabrescione B, thereby circumventing the pro-metastatic risks associated with direct STK38 inhibition. This strategy elicited robust anti-tumor responses, particularly in STK38-high models, and highlights a mechanism-informed therapeutic avenue for managing pRCC.

## Discussion

In this study, we uncovered a multifaceted role for STK38 in papillary renal cell carcinoma (pRCC), delineating its dual contributions to Hedgehog (Hh) pathway activation and tumor-associated chromatin release, ultimately linking it to the maintenance of stem-like tumor cell populations and tumor heterogeneity. Through a comprehensive series of in vitro, in vivo, and patient-derived organoid experiments, we identified STK38 as a critical modulator of tumor heterogeneity and demonstrated the therapeutic value of targeting its downstream effectors in STK38-high pRCC tumors.

Previous studies have reported context-dependent roles of STK38 across different cancers, with both tumor-promoting and suppressive functions described depending on signaling context. Our data suggest that STK38 promotes a self-renewing, undifferentiated tumor phenotype via selective activation of the Hh pathway, while also shaping the tumor microenvironment by modulating chromatin dynamics. As a highly conserved serine/threonine kinase, STK38 plays critical roles in developmental patterning, cellular differentiation, and organogenesis, and has been reported to intersect with key regulatory pathways involved in cell fate determination, including Wnt and Notch [19–23]. These interactions suggest that STK38 may function as a central regulatory hub coordinating multiple lineage-determining programs in tumor evolution.

The heterogeneity of pRCC, especially in type II tumors, is increasingly understood to reflect different stages of tumor progression [24, 25]. Recent single-cell analyses have proposed distinct molecular subtypes—such as the Tumor Programs (TP1/TP2) and more granular M1–M5 classifications—which differ in vascularization, metabolic activity, and pathway engagement [26, 27]. Notably, Hh, Wnt, Notch, and EMT signatures are differentially enriched across these subtypes, supporting our observation that STK38 may modulate the balance between Wnt and Hedgehog signaling in a context-dependent manner. In early-stage tumors, STK38 may inhibit Wnt signaling and suppress tumorigenesis. However, as malignancy progresses and the need for maintaining tumor self-renewal capacity increases, STK38-high subpopulations may become more prevalent. These high-STK38 cells may acquire the capacity to activate Hedgehog signaling through GSK3β and KIF7, thereby promoting the expansion of undifferentiated tumor subpopulations and enhancing tumor plasticity. Moreover, activation of the Hedgehog pathway reinforces its own activity via autocrine and paracrine loops—secreted Hh ligands may further stimulate the pathway in neighboring tumor cells within the microenvironment, establishing a feedforward cycle that supports tumor propagation and intratumoral heterogeneity [28].

This signaling rewiring model may also explain how tumors evade monotherapeutic targeting. As our data suggest, STK38 shifts the substrate preference of GSK3β, stabilizing GLI1 while continuing to suppress β-catenin. This cross-talk between competing pathways may underlie resistance to Wnt or Hh pathway inhibition alone, highlighting the need for network-informed therapeutic strategies that account for pathway compensation and plasticity.

Beyond its signaling functions, STK38 also regulates non-apoptotic chromatin release. We identified a phenomenon resembling tumor cell NETosis-like chromatin release (tNET release), which was markedly enhanced in STK38-deficient cells under stress. This form of chromatin expulsion has been proposed to remodel the tumor microenvironment and promote immune evasion and metastatic seeding. Notably, we observed that tNET release preferentially occurred in more differentiated, STK38-low subpopulations, suggesting a functional cooperation between phenotypically distinct tumor compartments. While STK38-high, self-renewing cells may initiate long-term colonization, STK38-low cells could contribute to microenvironmental remodeling and immune evasion through tNET-associated chromatin structures during collective dissemination [29].

This duality carries important translational implications: although STK38 suppression may limit the self-renewal capacity of tumor-initiating cells, it may also promote a pro-metastatic microenvironment by triggering tNET-associated chromatin release. Therefore, direct inhibition of STK38 may carry unintended risks. In light of this, we focused on its downstream effector GLI1, using the small-molecule inhibitor Glabrescione B (GlaB). GlaB showed robust anti-tumor activity, particularly in STK38-high tumors, and promoted apoptotic responses without overt toxicity. Our findings are consistent with prior reports of STK38 enhancing cellular sensitivity to stress-induced apoptosis [30–33], suggesting that GLI1 inhibition may synergize with STK38-driven stress pathways to induce more effective tumor cell death.

Furthermore, we identified a transcriptional positive feedback loop in which GLI1 directly binds to the STK38 promoter and enhances its expression. This autoregulatory circuit may sustain a self-renewing tumor subpopulation and contribute to intratumoral stability, therapeutic resistance, and disease recurrence. Targeting this axis at the level of GLI1 provides a focused strategy to disrupt this regenerative loop without broadly impairing essential signaling pathways.

This study underscores the importance of mechanism-informed targeting in heterogeneous malignancies. Rather than broadly suppressing a master regulator like STK38—which exerts divergent effects across cell populations—targeting its downstream effectors allows for selective disruption of oncogenic outputs while preserving homeostatic functions. Our findings also underscore the therapeutic potential of targeting context-specific vulnerabilities within the tumor ecosystem, including chromatin-based immune evasion and phenotypic cooperation between distinct tumor subpopulations during metastasis.

Nonetheless, several limitations remain. While we demonstrated functional interactions between STK38 and both KIF7 and GSK3β, residue-level validation of binding interfaces remains to be completed. Future studies employing site-directed mutagenesis, live-cell imaging, and spatial multi-omics will be essential to fully define STK38’s regulatory logic and its dynamic role in tumor evolution. Moreover, evaluating combinatorial strategies that integrate GLI1 inhibition with immune checkpoint blockade [34] or that selectively impair self-renewing tumor populations may represent a rational next step in pRCC therapy development.

In conclusion, our work establishes STK38 as a central coordinator of cellular plasticity and intratumoral heterogeneity in pRCC, and identifies GLI1 inhibition as a promising means of disrupting oncogenic self-renewal programs in aggressive tumor subsets.

## Materials and methods

### Bioinformatics analysis

Transcriptomic data from three GEO datasets (GSE15641, GSE152938, and GSE188486), including various renal cancer subtypes and normal tissues, were analyzed using the limma package in R. Genes with adjusted *p* < 0.05 and |log₂FC| > 0.5 were considered significantly differentially expressed. The results were further validated using the TCGA-KIRP cohort. Gene set enrichment analysis (GSEA) was conducted using TCGA transcriptomic profiles, with samples stratified based on STK38 expression. Gene Ontology (GO) analysis was performed to identify enriched biological processes. For single-cell RNA sequencing analysis, data from GSE152938 were processed using Seurat-based unsupervised clustering to identify distinct cellular clusters. Feature plots were generated using Seurat and visualized with ggplot2 to assess the relationship between STK38 expression and stemness-associated markers. In the Supplementary Materials, we provide the full list of differentially expressed genes across the datasets as part of the Supplementary Table 1 Differential Expression Analysis Gene Lists file.

### Western blotting

Cells were lysed in RIPA buffer (Beyotime, China) supplemented with protease and phosphatase inhibitors for standard western blotting procedures. For co-immunoprecipitation (co-IP) assays, cells were lysed using NP-40-based lysis buffer (Beyotime) on ice for 30 min. Protein concentrations were quantified using the BCA assay, and equal amounts of lysate were resolved on SDS-PAGE gels and transferred to PVDF membranes. Membranes were blocked in 5% non-fat milk and incubated overnight at 4 °C with primary antibodies.

The following primary antibodies were used: anti-STK38 (Proteintech, #55335-1-AP; Abnova, Lot# H6121-0195), anti-KIF7 (Proteintech, #24693-1-AP), anti-MICAL1 (Proteintech, #14818-1-AP), anti-SHH (Proteintech, #20697-1-AP), anti-GLI1 (Invitrogen, #PA5-72942), anti-cleaved caspase-3 (CST, #9661), anti-caspase-3 (CST, #14220), anti-Histone H3 (HUABIO, #M1309-1), anti-PADI4 (Boster, #PB97272S), anti-citrullinated Histone H3 (Arg17) (CST, #97272), and anti-β-catenin (CST, #8480). For Hh pathway components, the Hedgehog Signaling Antibody Sampler Kit (CST, #26118T) was used.

Secondary HRP-conjugated anti-mouse or anti-rabbit antibodies (Proteintech) were applied for 1 h at room temperature. Bands were visualized using enhanced chemiluminescence reagents (Millipore) and quantified using ImageJ. Full, uncropped western blot images for all experiments are provided in the Supplementary File (“Uncropped Western Blot Images”).

### Co-immunoprecipitation–LC–MS/MS

Cells were lysed in ice-cold NP-40 buffer containing protease/phosphatase inhibitors. Clarified lysates were incubated with anti-STK38 (or isotype IgG control) at 4 °C overnight and captured on Protein A/G beads. After stringent washes, bound proteins were eluted, separated ∼1 cm by SDS-PAGE, and subjected to in-gel trypsin digestion. Peptides were analyzed by nanoLC coupled to a high-resolution Orbitrap mass spectrometer in data-dependent HCD mode. Raw files were searched with Sequest HT against the UniProt Homo sapiens database; carbamidomethyl (C) was set as a fixed modification and oxidation (M) /protein N-terminal acetylation as variable. FDR was controlled at 1% at the PSM and protein levels using Percolator. Contaminants and reverse hits were removed.

### Tumorsphere formation assay

ACHN, Caki-2, and A498 cells were seeded in ultra-low attachment 6-well plates at a density of 5000 cells/well in serum-free medium. ACHN cells were cultured in DMEM/F12, Caki-2 in McCoy’s 5A, and A498 in RPMI-1640, each supplemented with 20 ng/mL EGF, 20 ng/mL bFGF, 1× B27, and 1× N2. Spheres were maintained for up to 14 days, and images were acquired on days 0, 3, 7, and 14. Only spheroids with a diameter greater than 75 μm were counted under an inverted microscope. Tumorsphere formation efficiency was calculated as the number of spheres formed per 1000 seeded cells. Cell lines were regularly tested for mycoplasma contamination using PCR and authenticated by short tandem repeat profiling.

### Extreme-limiting dilution assay

Fluorescence-activated cell sorting was used to sort live tumor cells into 24-well ultra-low attachment plates (Corning). Each well was seeded with 50, 100, or 200 cells in a sphere-forming medium composed of serum-free DMEM/F12 GlutaMAX (Gibco), McCoy’s 5A (Pricella), 20 ng/mL EGF, 10 ng/mL bFGF, B27 supplement, and N2 supplement (both from Gibco). Plates were centrifuged at 300 × *g* for 5 min to improve single-cell distribution, and empty wells were excluded from further analysis. After 7–10 days of culture, wells were scored for sphere formation (positive or negative). The resulting limiting dilution data (cell dose, number of wells tested, and number of positive wells) were analyzed in R 4.3.2 using the statmod package, following the method of Hu and Smyth [35], to estimate stem cell frequencies and 95% confidence intervals.

### Microscale thermophoresis

MST was conducted using the NanoTemper Monolith Pico system. KIF7 was fluorescently labeled using a RED-NHS 2nd Generation labeling kit (NanoTemper). Labeled KIF7 was held at a constant concentration (50 nM), and STK38 was titrated in a 1:2 dilution series across 18 capillaries (starting at 10 µM). Samples were incubated for 15 min at room temperature in MST buffer (20 mM HEPES, 150 mM NaCl, 0.05% Tween-20, pH 7.4), loaded into premium capillaries, and thermophoresis was measured at 25 °C. Binding curves were analyzed using MO. Affinity Analysis software. The dissociation curve showed concentration-dependent binding, confirming a specific interaction between STK38 and KIF7.

### Dual-luciferase reporter assay and site-directed mutagenesis

Promoter fragments of *STK38* (−2000 bp, −1000 bp, and −500 bp relative to TSS) were PCR-amplified from human genomic DNA and cloned into the pGL3-basic luciferase vector (Promega). Site-directed mutagenesis of individual GLI1-binding sites and the triple-site mutant was generated using a Fast Mutagenesis Kit (Vazyme) following the manufacturer’s instructions. All constructs were sequence-verified.

ACHN cells were co-transfected with 200 ng of pGL3-promoter constructs and 20 ng of pRL-TK Renilla luciferase plasmid (Promega) as internal control. After 36 h, luciferase activity was measured using the dual-luciferase Reporter Assay System (Promega) on a SpectraMax iD5 plate reader. Firefly luciferase activity was normalized to Renilla luciferase activity.

### Organoid culture and passaging

Establishment of RCC-derived organoids was performed as previously described with minor modifications [27]. Fresh tumor tissues from pRCC patients were collected and processed for organoid derivation. Samples were finely minced and enzymatically digested using a tumor-specific tissue dissociation solution (MB-0818L05S; Mobio) at 37 °C in a humidified incubator. The dissociation buffer was added at a volume of 25–50 times the tissue volume.

The digestion duration ranged from 30 to 120 min depending on tissue type and integrity. Progress was monitored microscopically every 10–15 min. The digestion was considered complete when a majority of the suspension consisted of single cells and clusters <70 μm. To terminate the enzymatic reaction, 2–5% FBS was added to the suspension, followed by gentle pipetting.

Cells were collected by centrifugation at 200 × *g* for 5 min, resuspended in TrypLE Express (DPBS + 1 mM EDTA; Thermo Fisher), and incubated at 37 °C for 5 min. Enzymatic activity was neutralized by the addition of AdDMEM/F12 containing 20% FBS. After a second centrifugation, the pellet was triturated 10 times to enhance mechanical dissociation and passed through a 70-μm cell strainer. The filtered single-cell suspension was resuspended in ice-cold organoid culture medium and mixed 3:1 with cold Matrigel (Corning). Approximately 20,000 cells per 40 μL Matrigel droplet were plated in pre-warmed 6-well plates, inverted for 10 min at 37 °C to solidify, and then overlaid with RCC organoid medium.

### Organoid maintenance and passaging

Organoids were passaged every 2–3 weeks at a ratio of 1:2 to 1:3. Y-27632 (10 μM) was included during initial plating and removed on day 7. For passaging, organoids were collected and treated with TrypLE Express containing 10 μM Y-27632 at 37 °C for 5 min, followed by neutralization with AdDMEM/F12 + 20% FBS. After centrifugation at 200 × *g*, organoids were pipetted in fresh medium to ensure fragmentation, and embedded again in Matrigel for reseeding.

### Animal experiments

All animal procedures were approved by the Laboratory Animal Center of Xiamen University (approval number: XMULAC20200039) and conducted in accordance with the ARRIVE 2.0 guidelines, the Declaration of Helsinki, and the NIH “Guide for the Care and Use of Laboratory Animals” (8th edition).

Male BALB/c-nu mice (6–8 weeks old) were housed under specific pathogen-free conditions with a 12-h light/dark cycle and ad libitum access to food and water. Mice were randomly assigned to treatment and control groups using a computer-generated randomization schedule. Tumor volumes were measured every 3 days by an investigator blinded to group allocation. No animals were excluded from the analysis, and no unexpected adverse events were observed. Sample sizes were based on previous studies and feasibility; no formal power analysis was conducted.

### Ethics statement

All animal procedures were approved by the Laboratory Animal Center of Xiamen University (approval number: XMULAC20200039) and were conducted in accordance with the ARRIVE 2.0 guidelines, the Declaration of Helsinki, and the NIH “Guide for the Care and Use of Laboratory Animals” (8th edition). For the use of human tissue samples, ethical approval was obtained from the Medical Ethics Committee of Xiamen University (approval number: XDYX202411K73). Informed consent was obtained from all participants or their legal guardians prior to sample collection.

## Supplementary information

Supplementary Figure Legends and Table Footnotes

Supplementary Figure 1

Supplementary Figure 2

Supplementary Figure 3

Supplementary Table 1. Differential expression analysis gene lists

Supplementary Table 2. Extreme limiting dilution data

Supplementary Table 3. Hedgehog-related proteins identified by STK38 IP-MS

Uncropped Western Blot Images

## Data Availability

The datasets generated and analyzed during the current study are available from the corresponding author, Prof. Chen Shao, upon reasonable request. Publicly available RNA-seq datasets used in this study include GSE15641, GSE152938, and GSE188486 from the Gene Expression Omnibus (GEO) database and the TCGA database.
