# Supplementary material for: STK38-mediated feedback loop regulation of the hedgehog pathway governing tumor heterogeneity in renal papillary carcinoma
Source: Cell Death Dis. 2026 Jan 15;17(1):38. doi: 10.1038/s41419-025-08225-4 (PMC12808802; doi:10.1038/s41419-025-08225-4)
Supplement: Supplementary file 1 — Supplementary Figure Legends and Table Footnotes [file 41419_2025_8225_MOESM1_ESM.docx]

**Supplementary figure legends and table footnotes**

FigureS1.

(a) Expression levels of MICAL1 across three RCC subtypes (ccRCC, pRCC, chRCC) in the TCGA-KIRC, -KIRP, and -KICH datasets. MICAL1 was most highly expressed in pRCC with statistically significant differences among subtypes.

(b) Comparison of MICAL1 expression in tumor versus adjacent non-tumor tissues within the TCGA-KIRP cohort.

(c) MICAL1 expression in normal tissues, lymph node-negative (N0), and lymph node-positive (N1) pRCC patients. Expression was significantly elevated in both N0 and N1 groups compared to normal controls.

(d) Expression levels of *STK38* across three major RCC subtypes (ccRCC, pRCC, chRCC) in TCGA datasets. *STK38* was most highly expressed in pRCC, with statistically significant differences observed among subtypes.

(e) *STK38* expression in tumor versus adjacent normal tissues from pRCC patients in the TCGA-KIRP cohort, showing significant upregulation in tumors.

(f) *STK38* expression in normal tissue, lymph node-negative (N0), and lymph node-positive (N1) pRCC patients. *STK38* was significantly elevated in both N0 and N1 groups compared to normal controls.

(g) Gene ontology (GO) analysis of differentially expressed genes between *STK38*-high and *STK38*-low pRCC tumors (top and bottom 40%, TCGA-KIRP). Enriched terms were related to stem cell maintenance, tumor initiation, epithelial cell development, and morphogenetic signaling, suggesting a potential role for STK38 in regulating intratumoral heterogeneity.

(h) Single-cell transcriptomic analysis of GSE152938 (pRCC) using Seurat identified 12 transcriptionally distinct cellular clusters.

(i) Feature plots showing the expression patterns of *STK38* and selected stemness-associated genes (ALDH1A1, CD44, ABCB1, NANOG, CD24) across clusters. *STK38* expression was detected in multiple tumor-associated clusters, partially overlapping with stemness markers, suggesting a potential association with regenerative tumor compartments.

Statistical significance was assessed by one-way ANOVA or unpaired t-test as appropriate. *P* < 0.05: *, *P* < 0.01: **, *P* < 0.001: ***.

FigureS2.

(a) Immunofluorescence images of ACHN cells overexpressing STK38 or empty vector (EV, pCMV), treated with 700 nM SAG to induce Hedgehog signaling. KIF7 localization at the ciliary tip was assessed.

(b) Quantification of KIF7-positive ciliary tips as a percentage of total ciliated cells. *STK38* overexpression significantly increased KIF7 ciliary accumulation (n > 100 cells per group, across five replicates).

(c) Phos-tag gel electrophoresis showing KIF7 phosphorylation status in CRISPR-Cas9–mediated *STK38*-knockout versus wild-type ACHN cells. STK38 loss markedly reduced the phosphorylated KIF7 band, indicating its role in KIF7 phosphorylation.

(d) Efficiency validation of three siRNA constructs targeting KIF7 by qRT-PCR and Western blot. The most efficient siRNA was selected for use in main figure experiments.

(e) Efficiency validation of three siRNA constructs targeting GSK3β. The most effective sequence was used in downstream rescue assays.

(f) Structural modeling of the STK38–KIF7 interaction based on AlphaFold3 prediction. STK38 is shown in deep blue and KIF7 in pale green. The interaction interface is highlighted (STK38: gold; KIF7: orange). Hydrophobic interaction sites (e.g., PHE-280, LEU-27, VAL-114) are depicted as blue spheres, and hydrogen bonds (e.g., ASP-110, LYS-109) are shown as black dashed lines. Visualization was performed using PyMOL.

Statistical significance was determined using Student’s t-test or one-way ANOVA. *P* < 0.05: *, *P* < 0.01: **, *P* < 0.001: ***.

FigureS3.

(a) Correlation analysis between *GLI1* and *STK38* expression across renal cancer samples in the TCGA pan-kidney dataset. A moderate positive correlation was observed (*P* < 0.001, r = 0.306), supporting GLI1 as a putative transcriptional regulator of *STK38*.

(b) DIC images of tumor spheres formed from control and *STK38*-knockdown ACHN cells under suspension culture. STK38-depleted cells frequently exhibited extracellular reticular structures surrounding the spheres, suggestive of chromatin release consistent with tNETosis-like features.

(c) Hematoxylin and eosin (H&E) staining of major organs (heart, liver, spleen, lung, kidney) from mice treated with Glabrescione B or vehicle control. Representative sections show no significant histopathological abnormalities. Scale bar, 100 μm.

**Supplementary Table 1.** Differential Expression Analysis Gene Lists

Gene lists from GSE15641, GSE152938, and GSE188486 (criteria: adj. P < 0.05, |log₂FC| > 0.5); overlaps used for candidate selection.

**Supplementary Table 2.** Extreme Limiting Dilution Data

Raw ELDA inputs (cell dose, wells tested/positive) and estimated stem-cell frequencies with 95% CIs.

**Supplementary Table 3.** Hedgehog-related proteins identified by STK38 IP-MS

Filtered Hedgehog-related proteins identified by STK38 IP-MS
