# Supplementary figures and images for: STK38-mediated feedback loop regulation of the hedgehog pathway governing tumor heterogeneity in renal papillary carcinoma
Source: Cell Death Dis. 2026 Jan 15;17(1):38. doi: 10.1038/s41419-025-08225-4 (PMC12808802; doi:10.1038/s41419-025-08225-4)

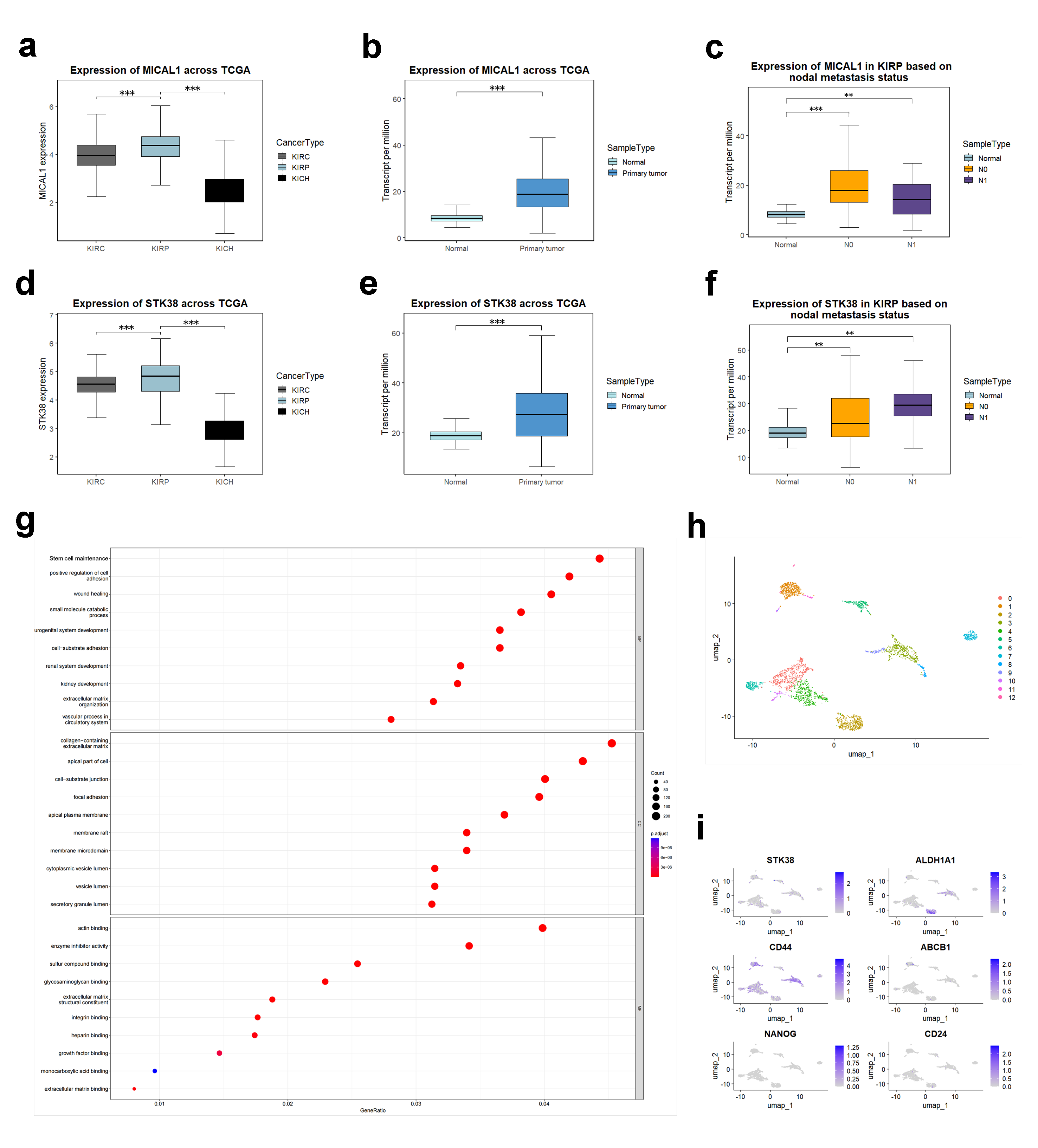

Supplement: Supplementary file 2 — Supplementary Figure 1 [file 41419_2025_8225_MOESM2_ESM.tif]

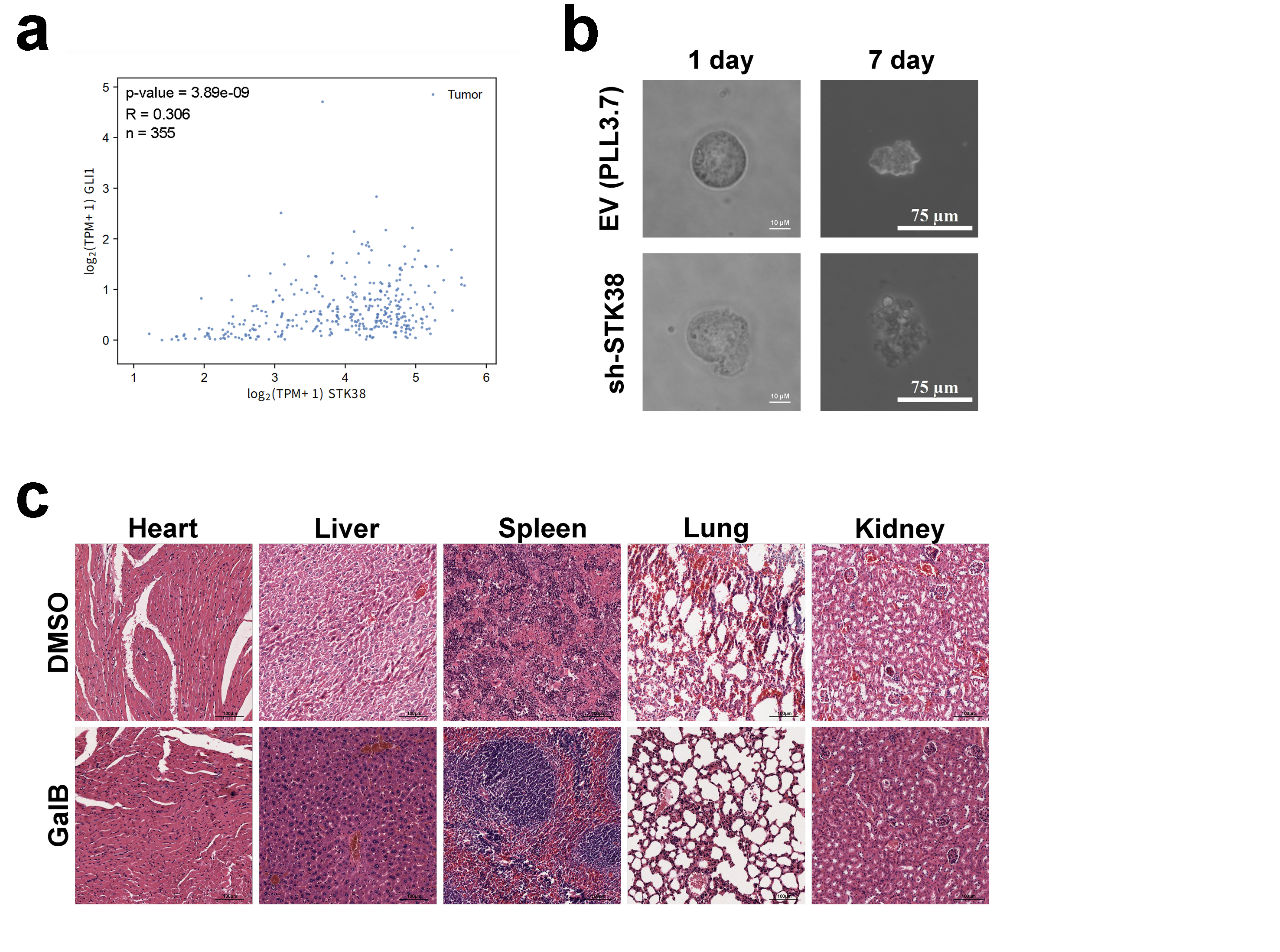

Supplement: Supplementary file 4 — Supplementary Figure 3 [file 41419_2025_8225_MOESM4_ESM.tif]
