# Supplementary material for: STK38-mediated feedback loop regulation of the hedgehog pathway governing tumor heterogeneity in renal papillary carcinoma
Source: Cell Death Dis. 2026 Jan 15;17(1):38. doi: 10.1038/s41419-025-08225-4 (PMC12808802; doi:10.1038/s41419-025-08225-4)

Supplementary File – Uncropped Blots

Pre-stained Protein Marker

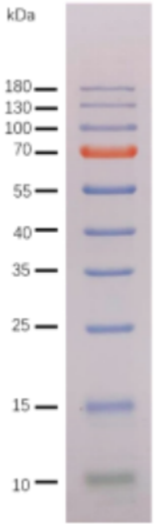

This is the pre-stained protein ladder used for all western blot experiments in this study.
The approximate molecular weights of the bands are:
~180 kDa, 130 kDa, 100 kDa, 70 kDa, 55 kDa, 40 kDa, 35 kDa, 25 kDa, 15 kDa, and 10 kDa.

Figure 2 – Uncropped blot of ACTB (Related to Figure 2d)

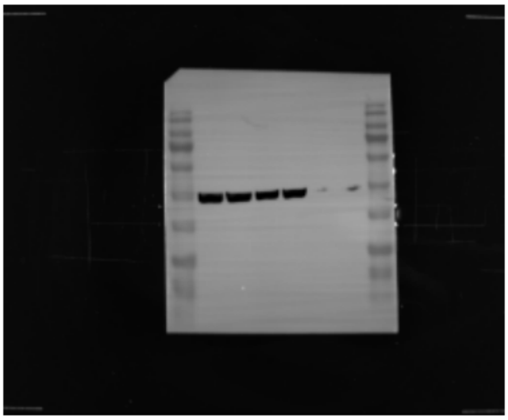

Figure 2 – Uncropped blot of GLI1 (Related to Figure 2d)

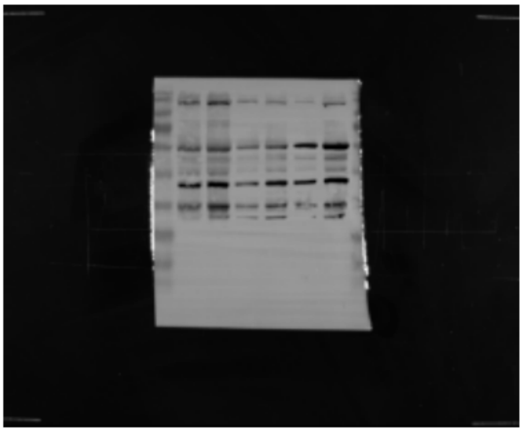

Figure 2 – Uncropped blot of STK38 (Related to Figure 2d)

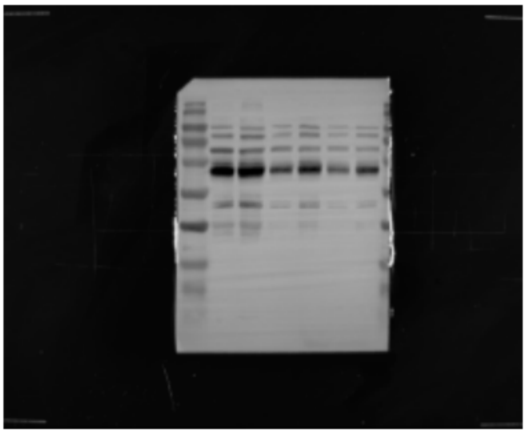

Figure 2 – Uncropped blot of Histone-H3 (Related to Figure 2d)

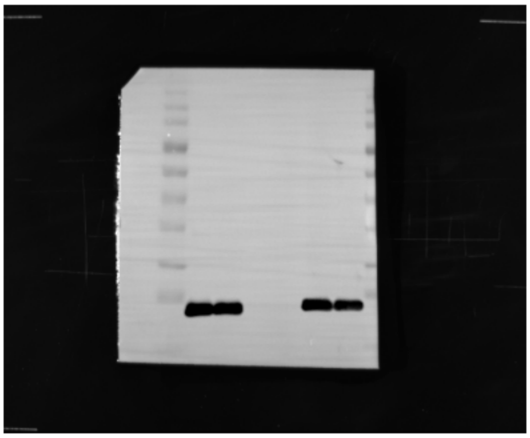

Figure 2 – Uncropped blot of ACTB (Related to Figure 2h)

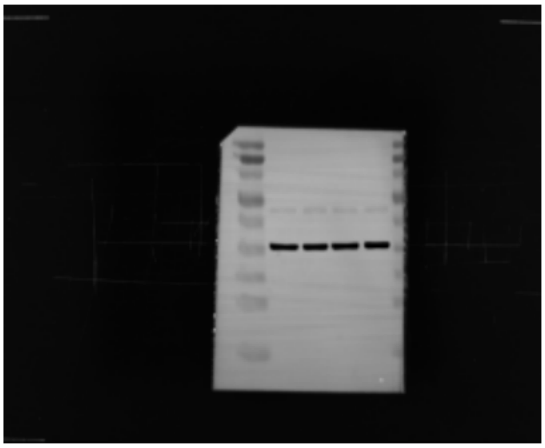

Figure 2 – Uncropped blot of GLI1 (Related to Figure 2h)

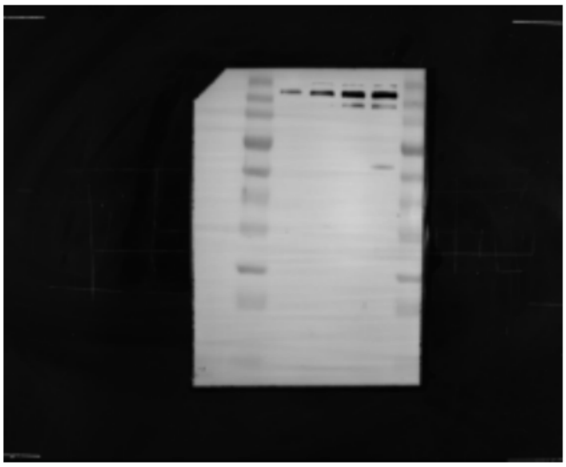

Figure 2 – Uncropped blot of PTCH1 (Related to Figure 2h)

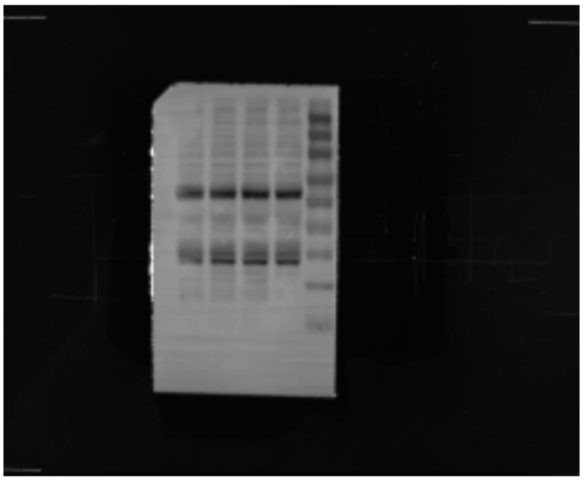

Figure 2 – Uncropped blot of SHH (Related to Figure 2h)

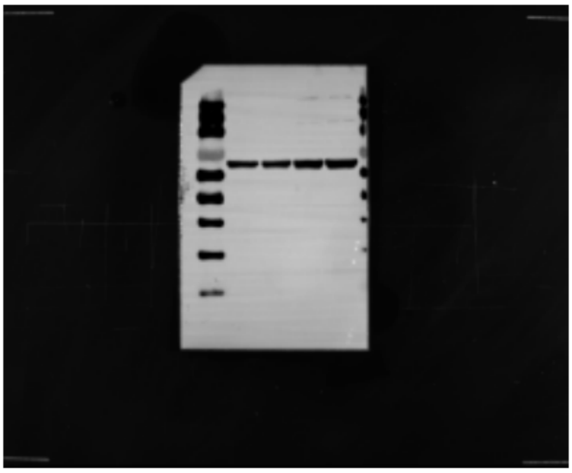

Figure 2 – Uncropped blot of STK38 (Related to Figure 2h)

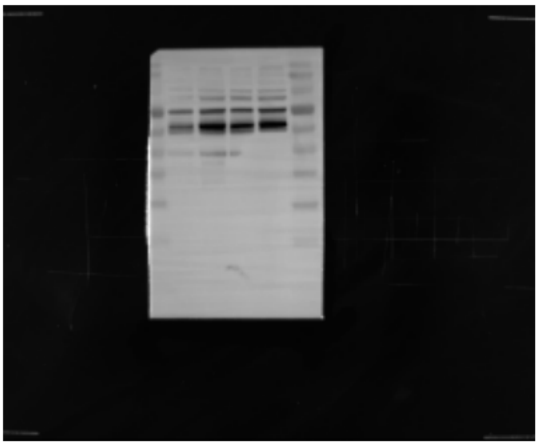

Figure 2 – Uncropped blot of ACTB (Related to Figure 2i)

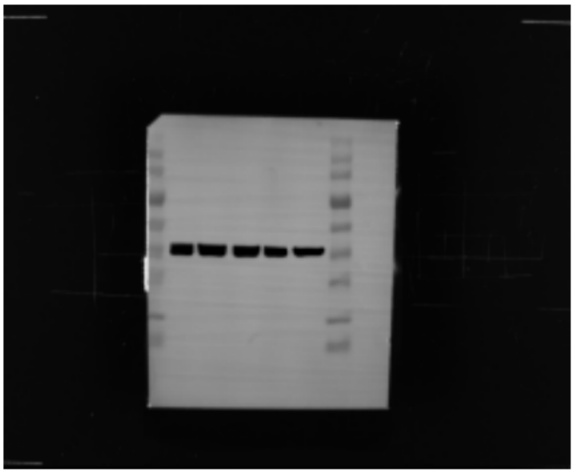

Figure 2 – Uncropped blot of GLI1 (Related to Figure 2i)

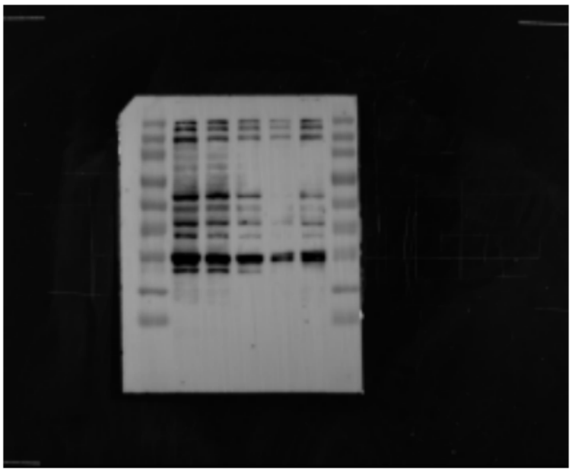

Figure 2 – Uncropped blot of SHH (Related to Figure 2i)

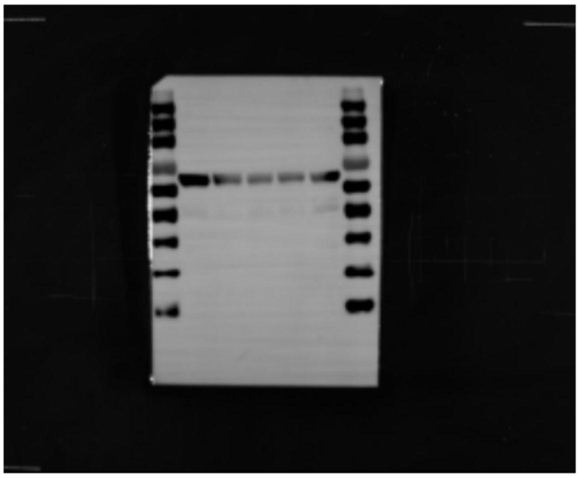

Figure 2 – Uncropped blot of STK38 (Related to Figure 2i)

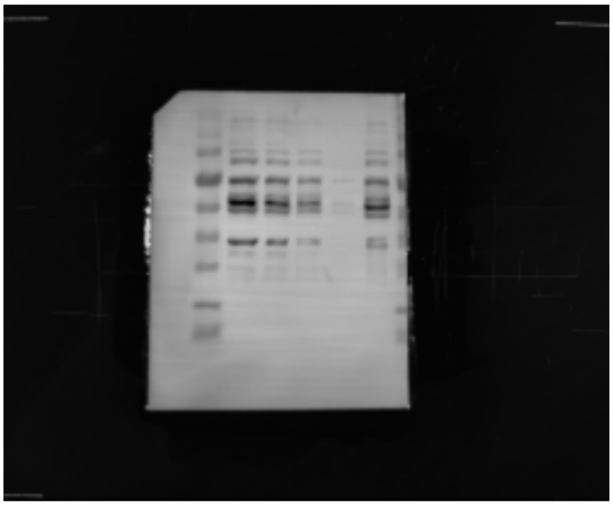

Figure 3 – Uncropped blot of GLI1 (Related to Figure 3a)

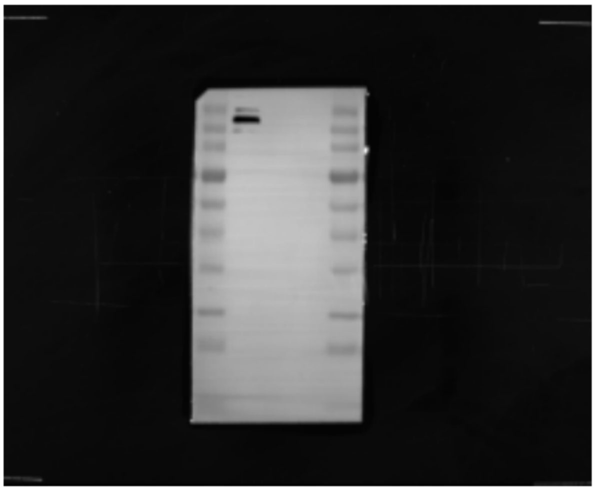

Figure 3 – Uncropped blot of GLI2 (Related to Figure 3a)

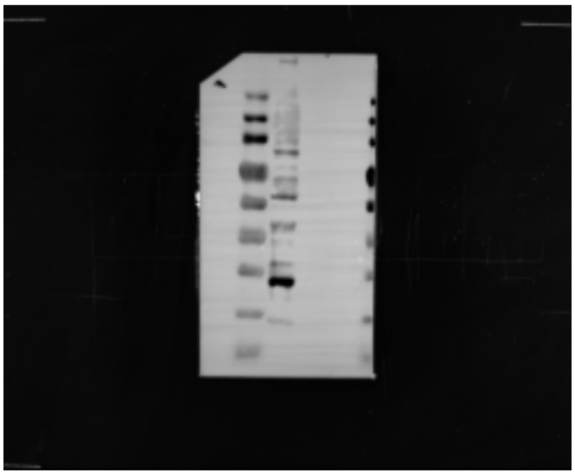

Figure 3 – Uncropped blot of GLI3 (Related to Figure 3a)

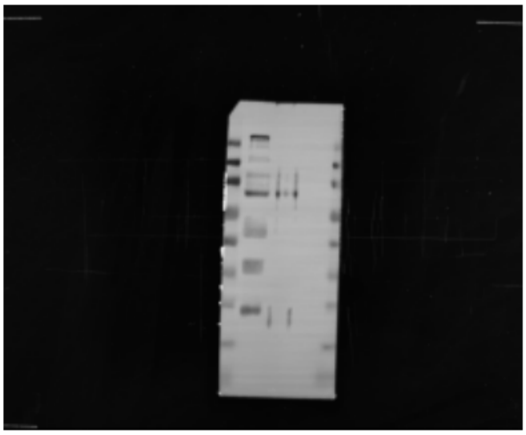

Figure 3 – Uncropped blot of GSK3 β (Related to Figure 3a)

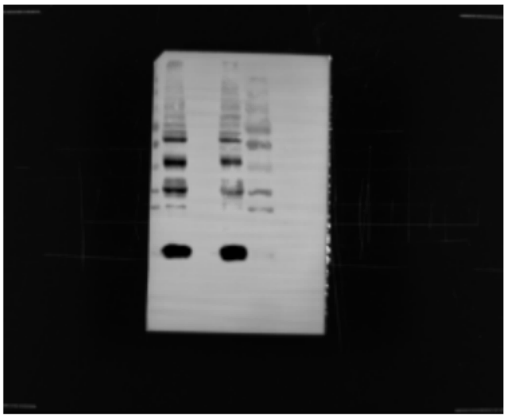

Figure 3 – Uncropped blot of KIF7 (Related to Figure 3a)

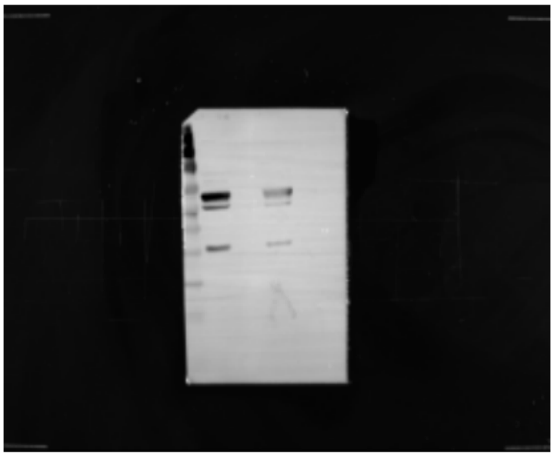

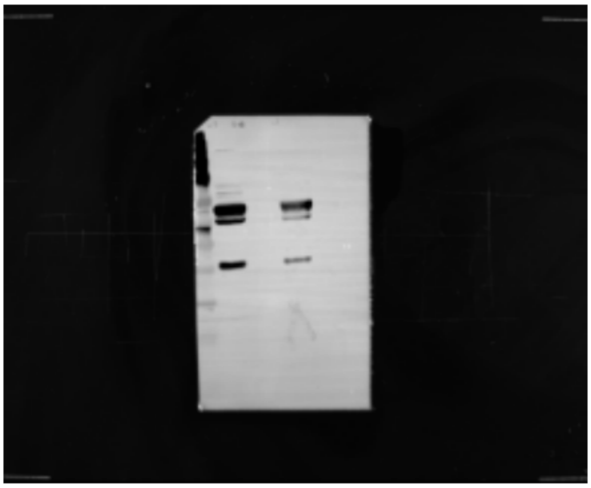

Figure 3 – Uncropped blot of PTCH1 (Related to Figure 3a)

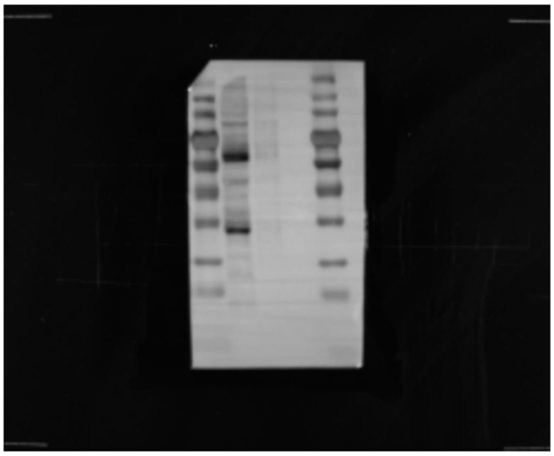

Figure 3 – Uncropped blot of PTCH2 (Related to Figure 3a)

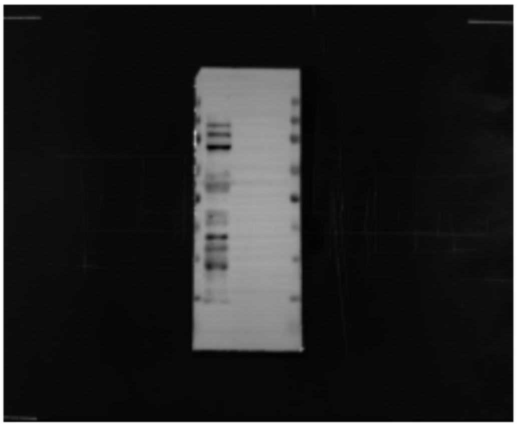

Figure 3 – Uncropped blot of SMO (Related to Figure 3a)

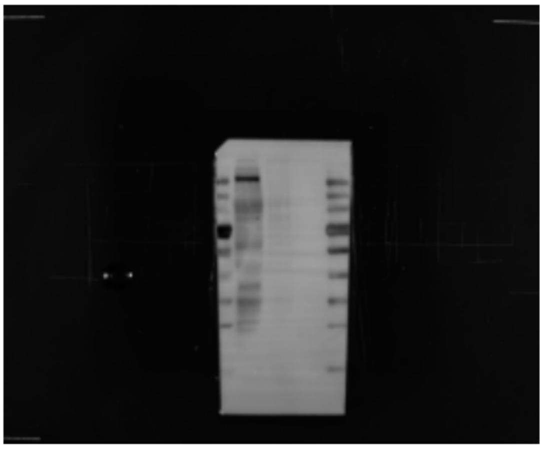

Figure 3 – Uncropped blot of STK38 (Related to Figure 3a)

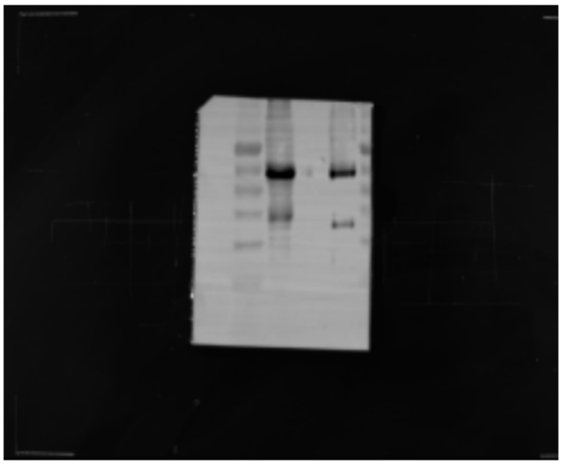

Figure 3 – Uncropped blot of GSK3 β (Related to Figure 3c)

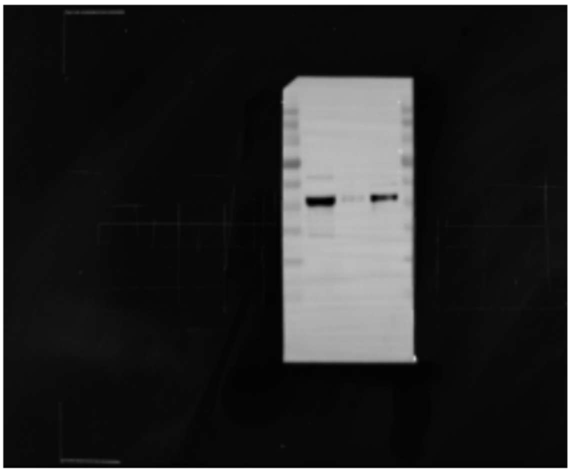

Figure 3 – Uncropped blot of STK38 (Related to Figure 3c)

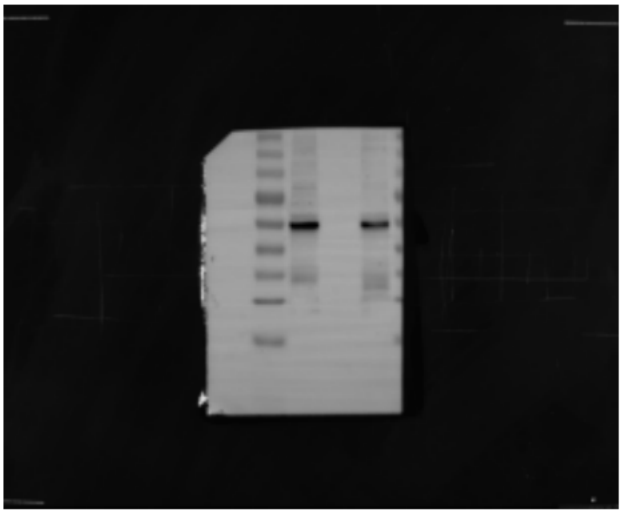

Figure 3 – Uncropped blot of KIF7 (Related to Figure 3c)

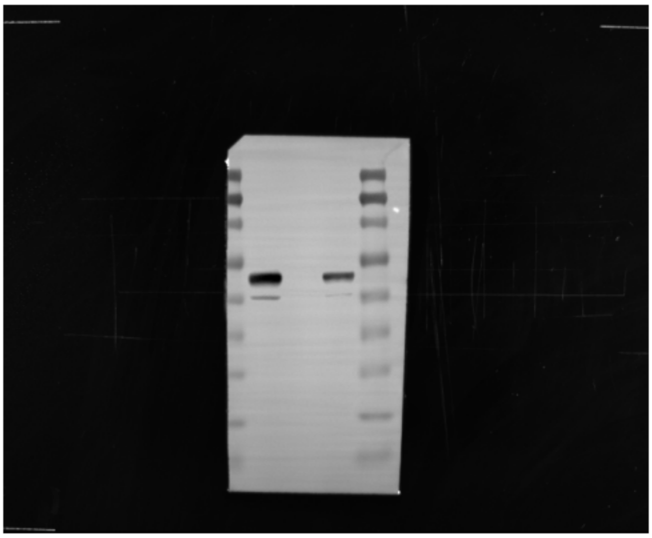

Figure 3 – Uncropped blot of STK38 (Related to Figure 3c)

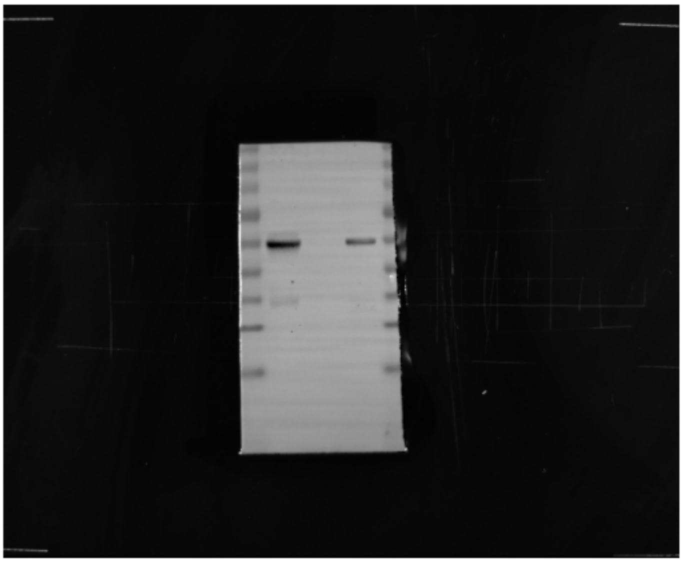

Figure 3 – Uncropped blot of KIF7 (Related to Figure 3d)

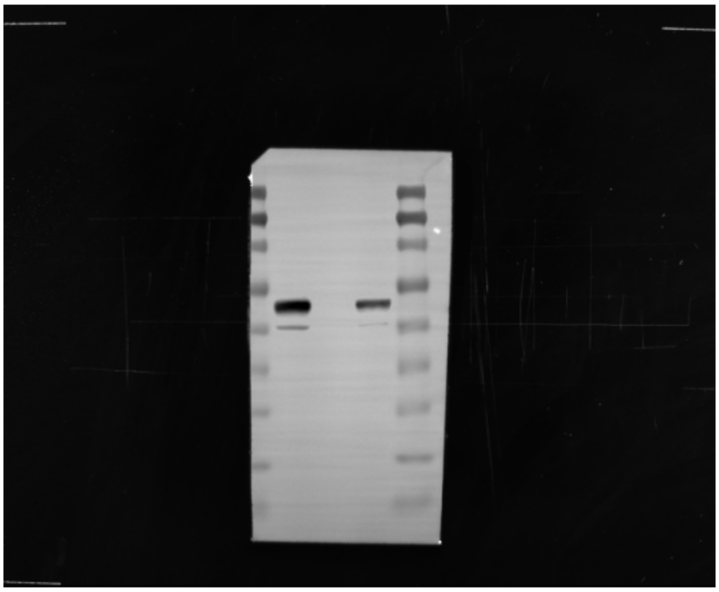

Figure 3 – Uncropped blot of STK38 (Related to Figure 3d)

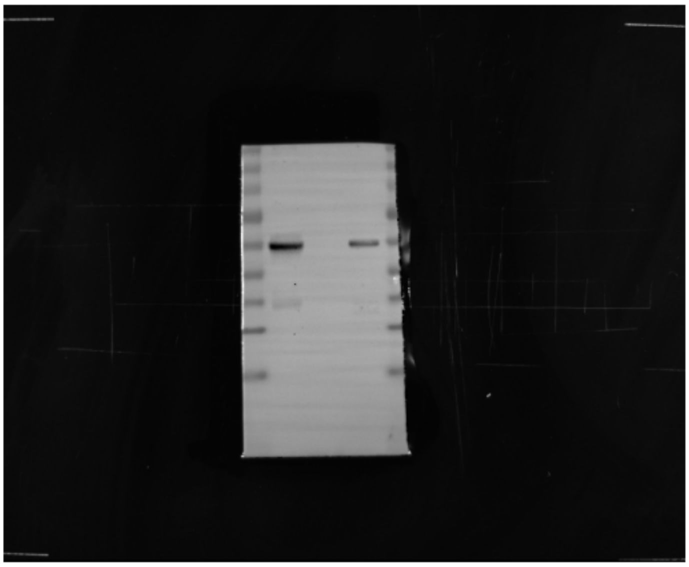

Figure 3 – Uncropped blot of GAPDH (Related to Figure 3f)

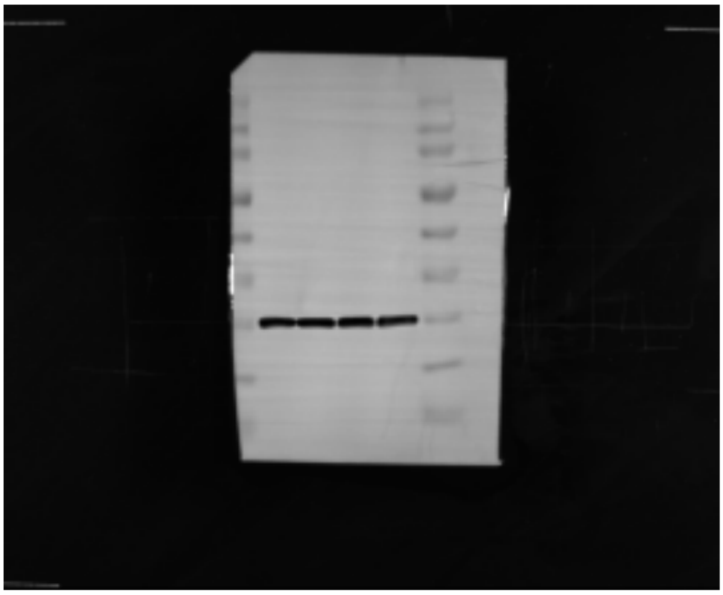

Figure 3 – Uncropped blot of Myc-tag (Related to Figure 3f)

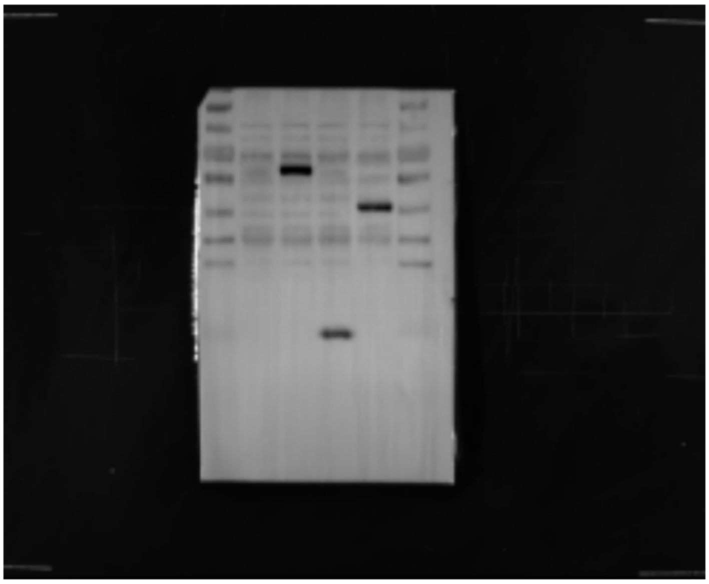

Figure 3 – Uncropped blot of STK38 (Related to Figure 3f)

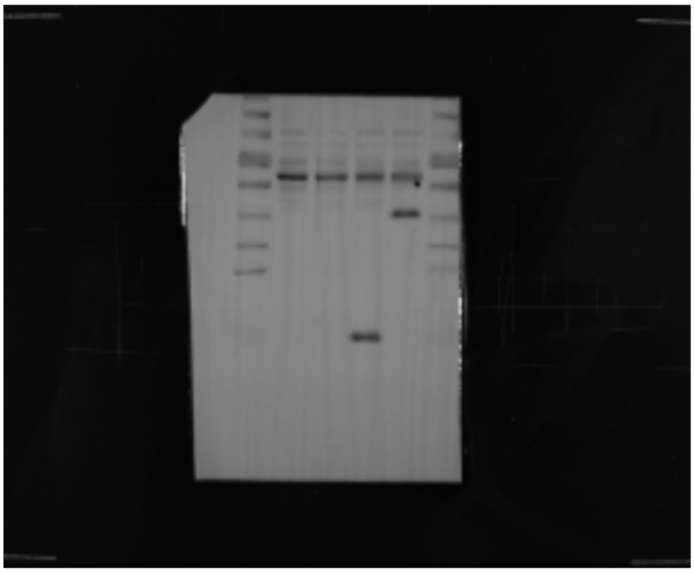

Figure 3 – Uncropped blot of ACTB (Related to Figure 3g)

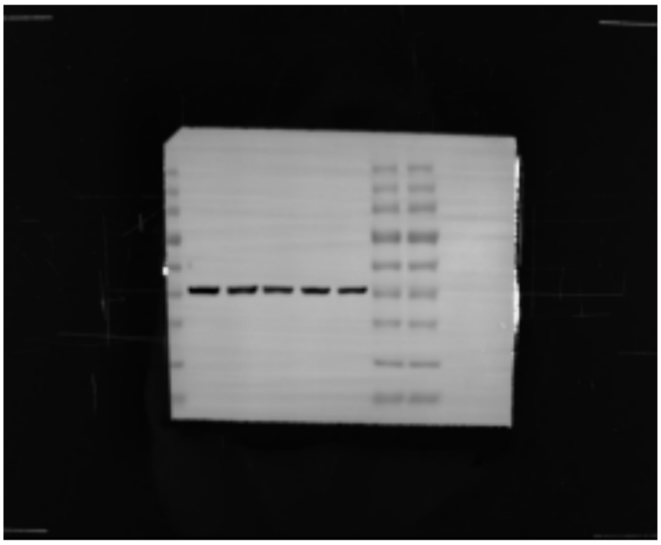

Figure 3 – Uncropped blot of β-catenin_Lysate (Related to Figure 3g)

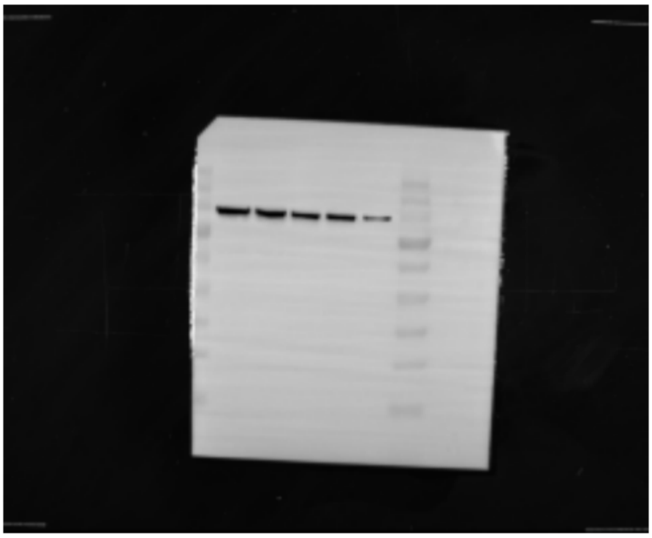

Figure 3 – Uncropped blot of β-catenin_IP (Related to Figure 3g)

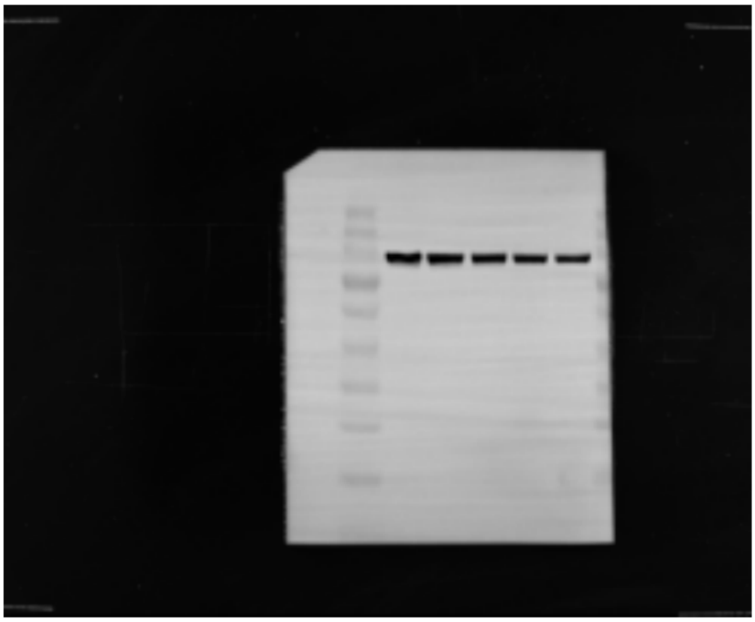

Figure 3 – Uncropped blot of GLI1_Lysate (Related to Figure 3g)

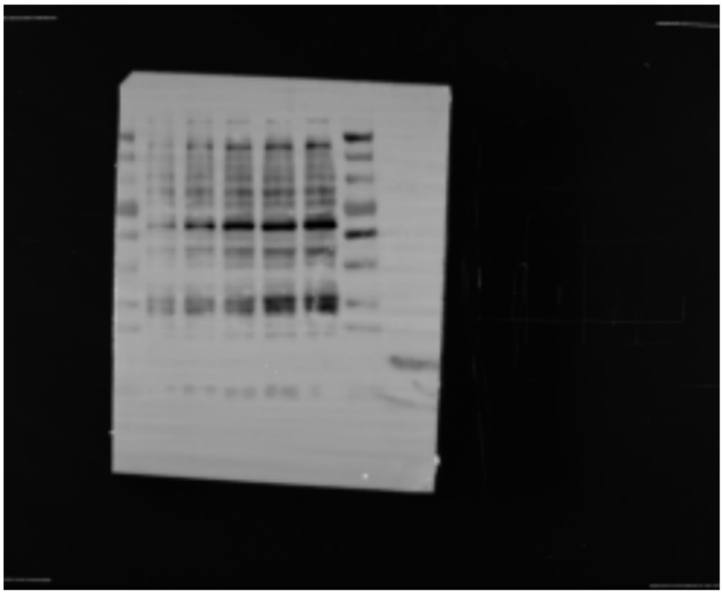

Figure 3 – Uncropped blot of GLI1_IP (Related to Figure 3g)

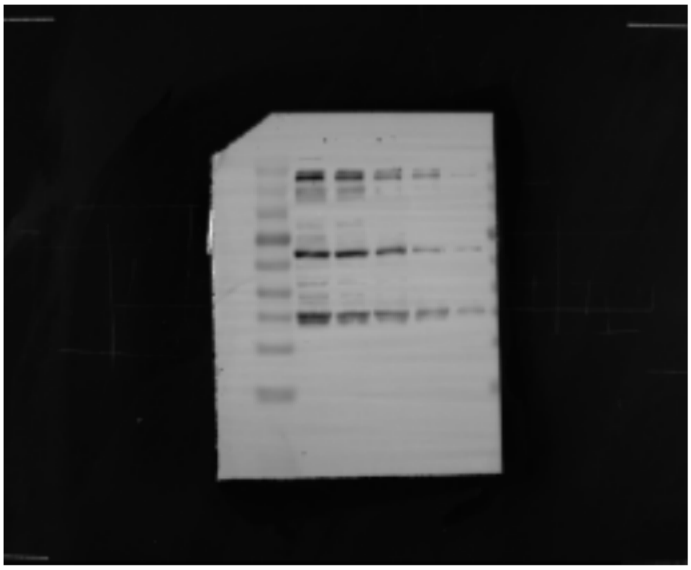

Figure 3 – Uncropped blot of GSK3 β _Lysate (Related to Figure 3g)

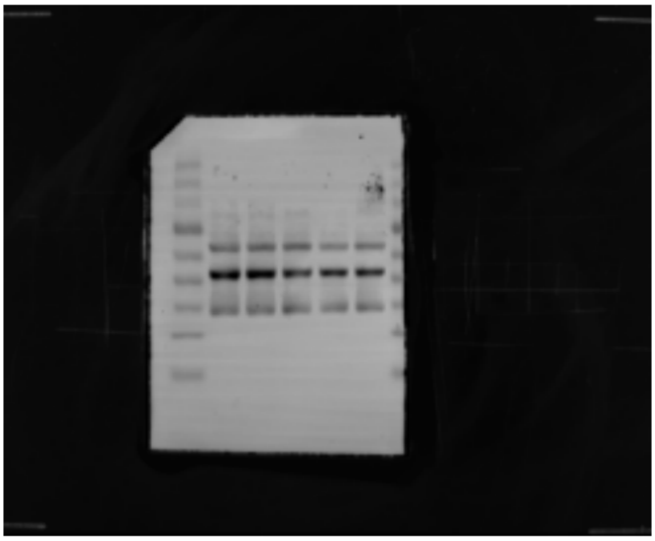

Figure 3 – Uncropped blot of GSK3 β -P_Lysate (Related to Figure 3g)

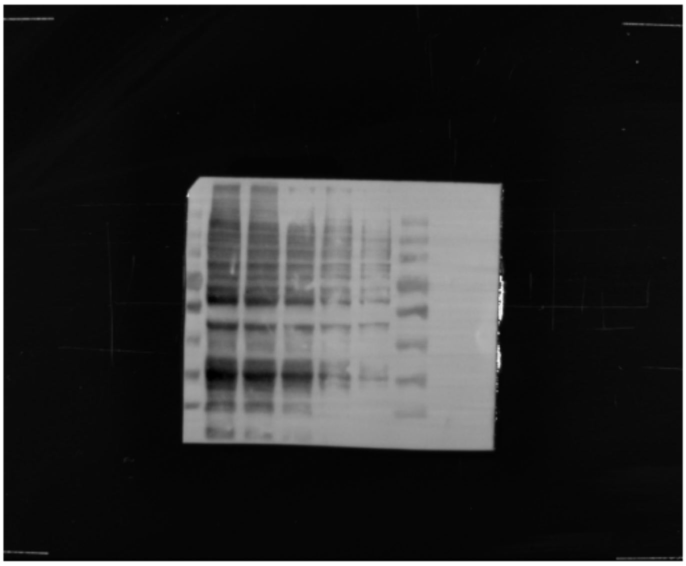

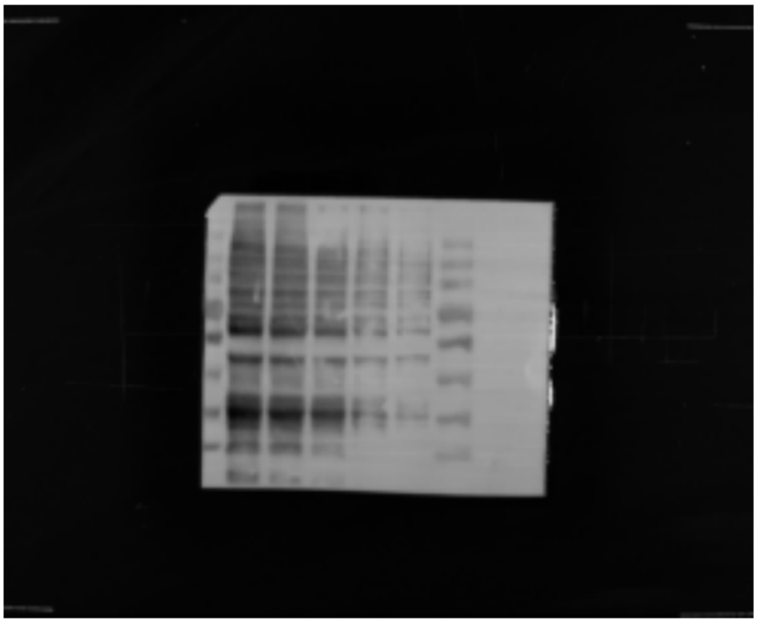

Figure 3 – Uncropped blot of STK38_IP (Related to Figure 3g)

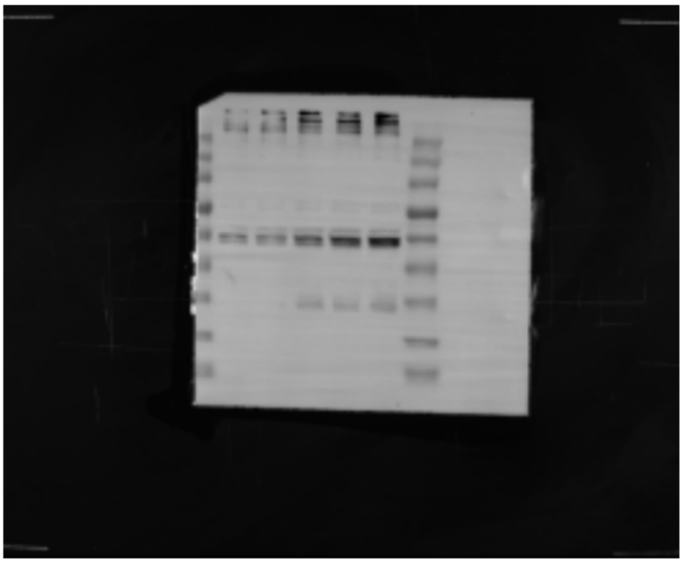

Figure 3 – Uncropped blot of STK38_Lysate (Related to Figure 3g)

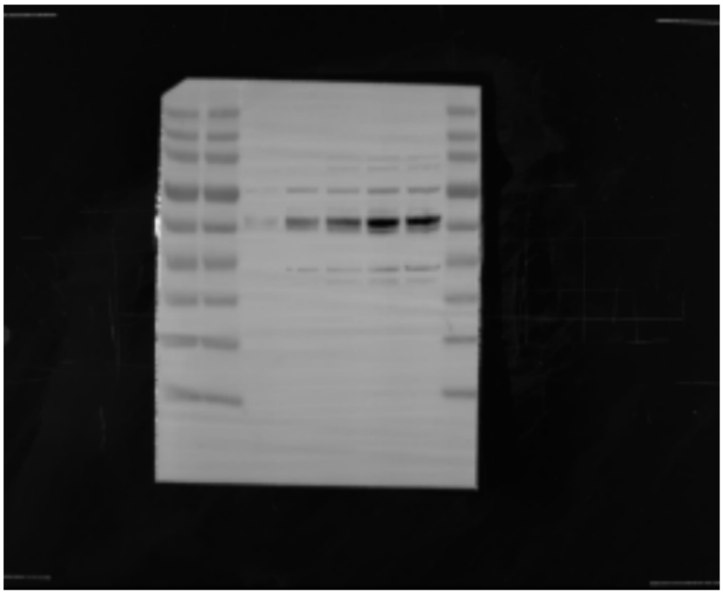

Figure 3 – Uncropped blot of β-catenin_Lysate (Related to Figure 3h)

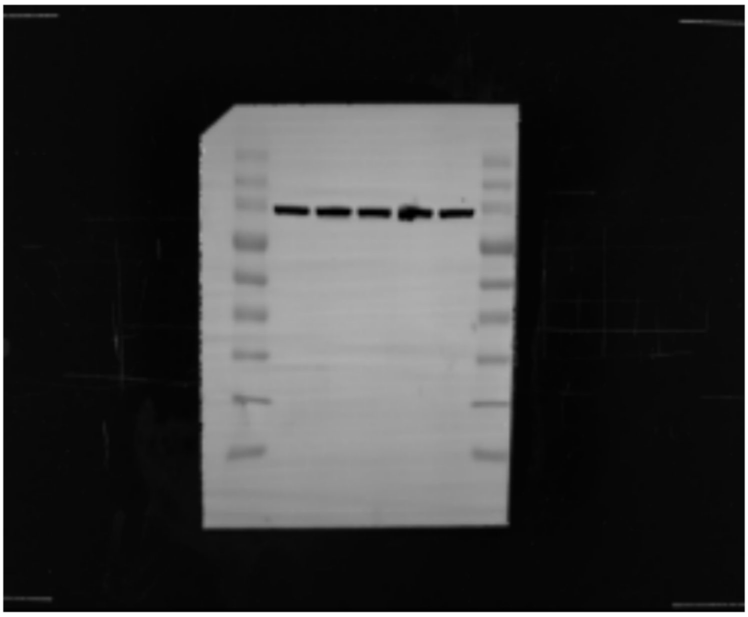

Figure 3 – Uncropped blot of β-catenin_IP (Related to Figure 3h)

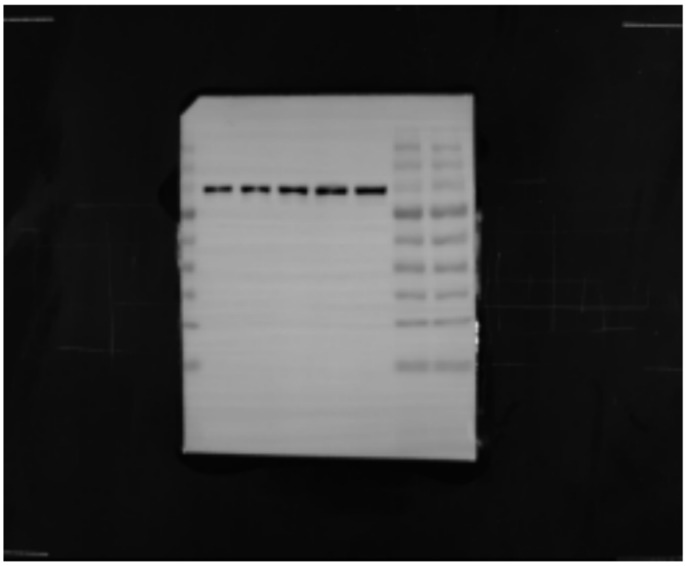

Figure 3 – Uncropped blot of GLI1_IP (Related to Figure 3h)

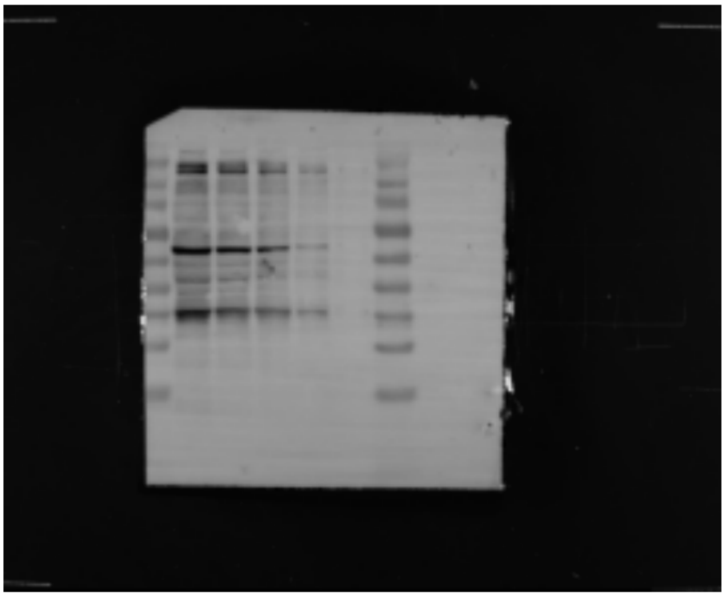

Figure 3 – Uncropped blot of STK38_IP (Related to Figure 3h)

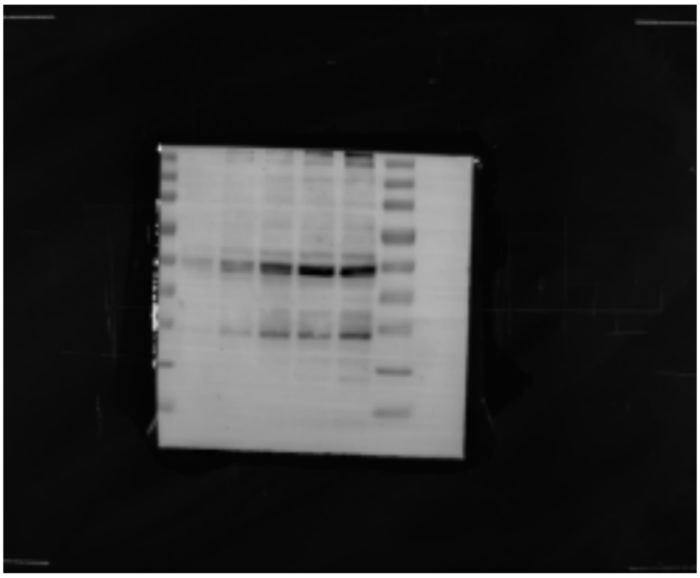

Figure 3 – Uncropped blot of ACTB (Related to Figure 3i)

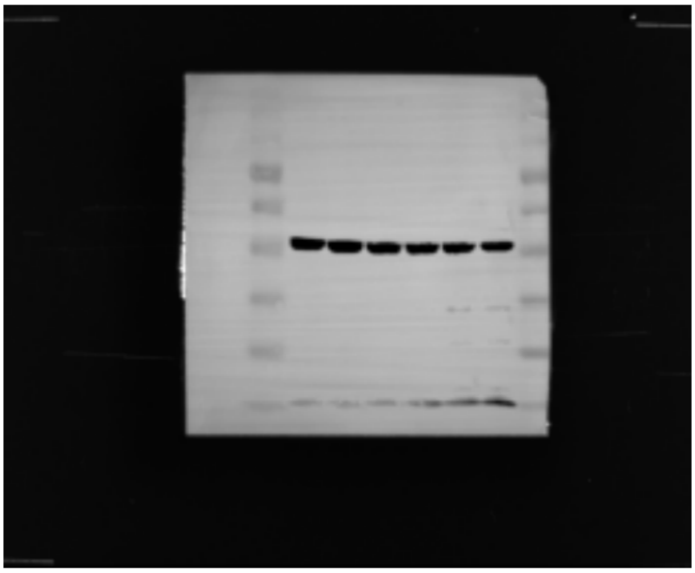

Figure 3 – Uncropped blot of β-catenin (Related to Figure 3i)

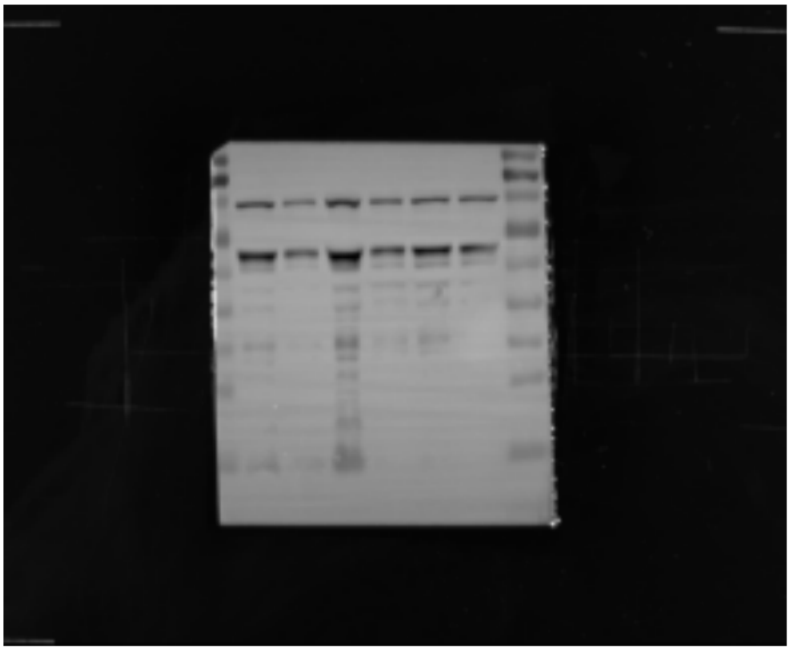

Figure 3 – Uncropped blot of GLI1 (Related to Figure 3i)

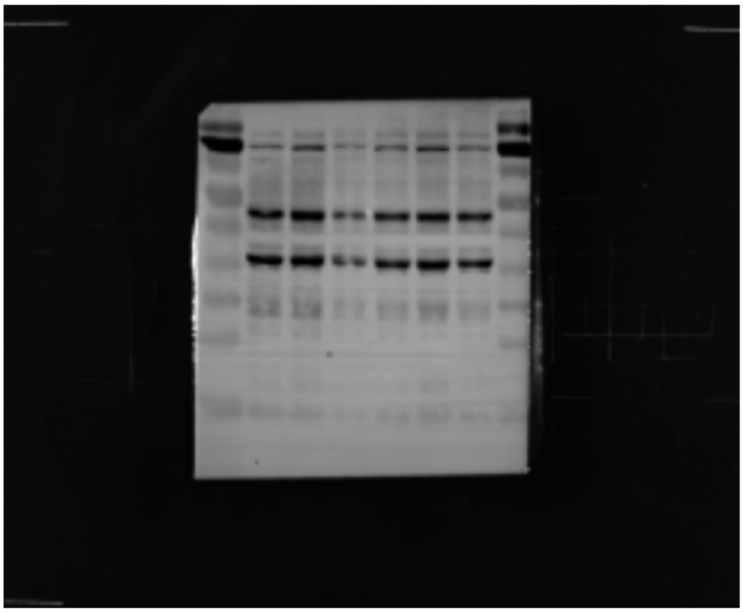

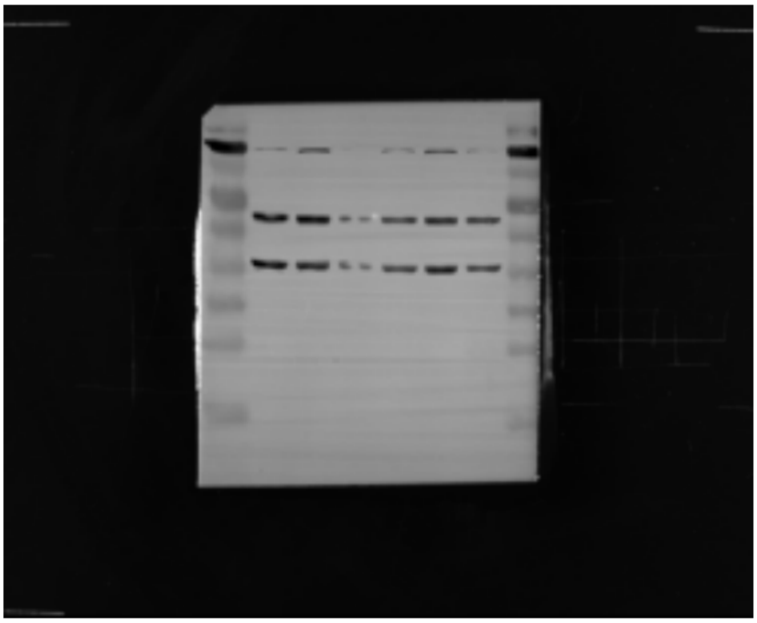

Figure 3 – Uncropped blot of GSK3 β (Related to Figure 3i)

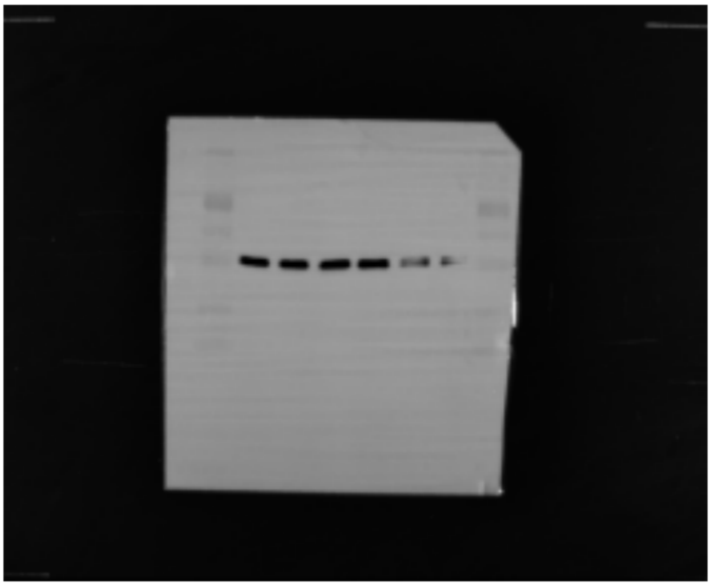

Figure 3 – Uncropped blot of KIF7 (Related to Figure 3i)

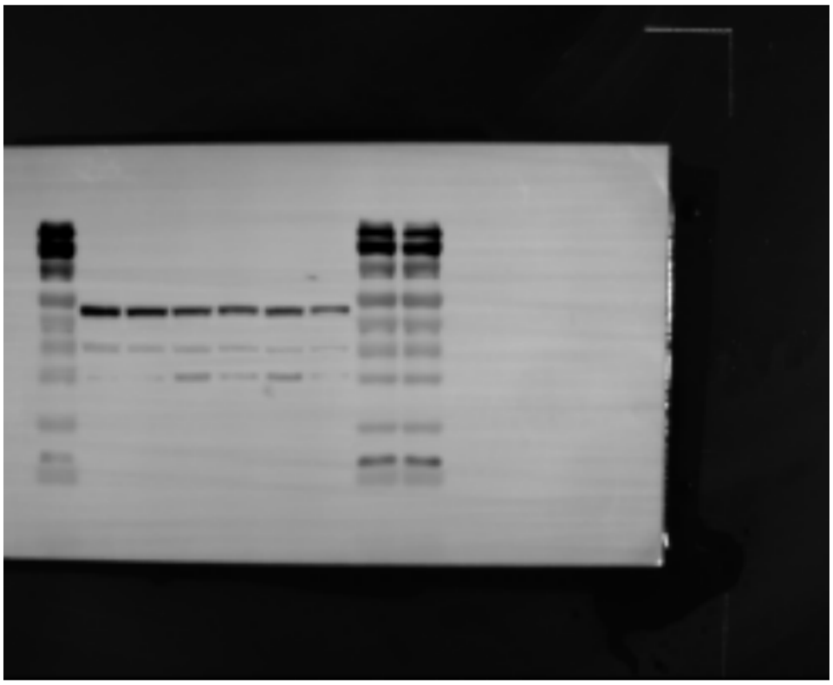

Figure 3 – Uncropped blot of SMO (Related to Figure 3i)

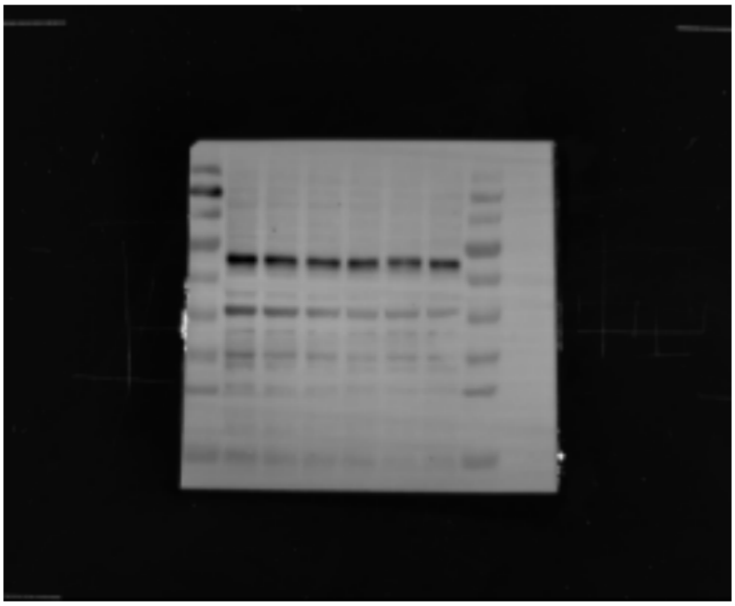

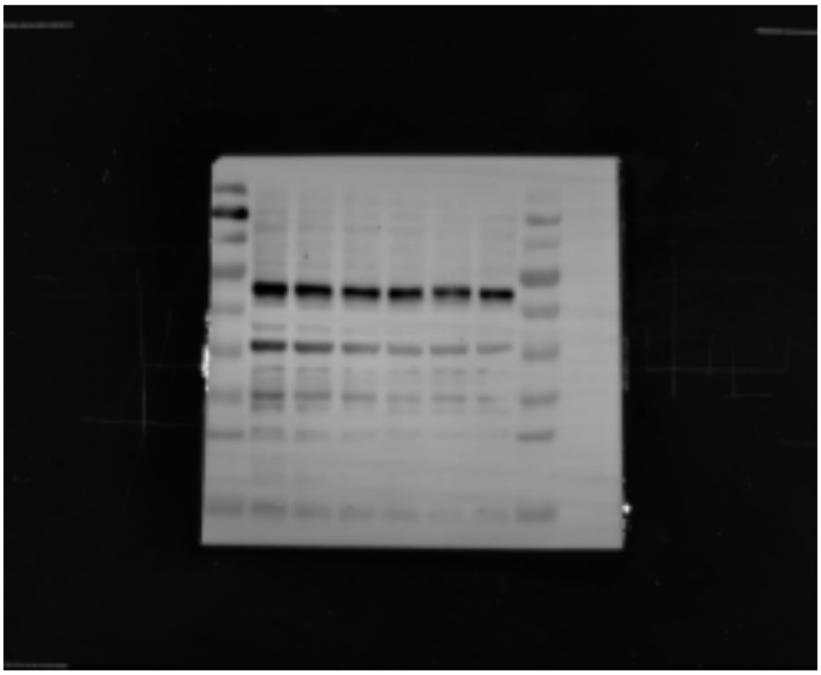
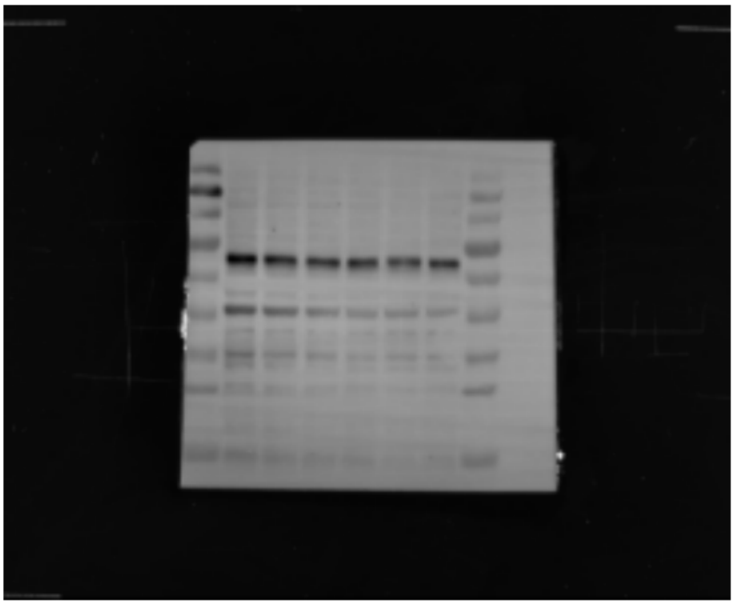

Figure 3 – Uncropped blot of STK38 (Related to Figure 3i)

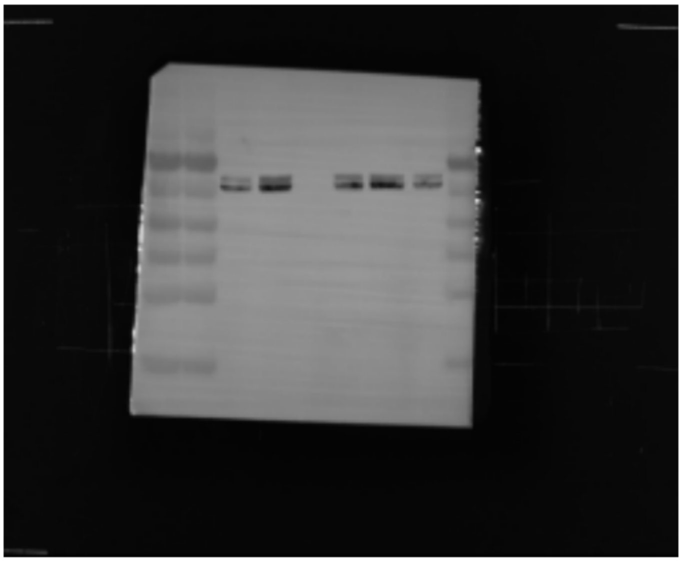

Figure 4 – Uncropped blot of ACTB (Related to Figure 4c)

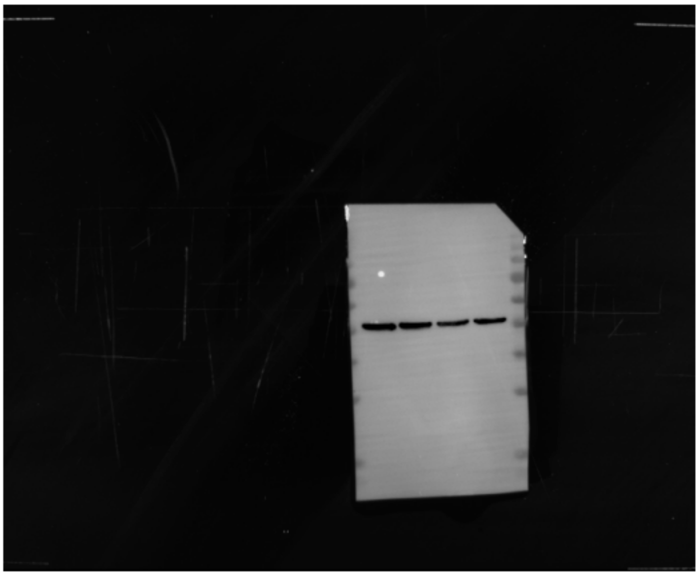

Figure 4 – Uncropped blot of GLI1 (Related to Figure 4c)

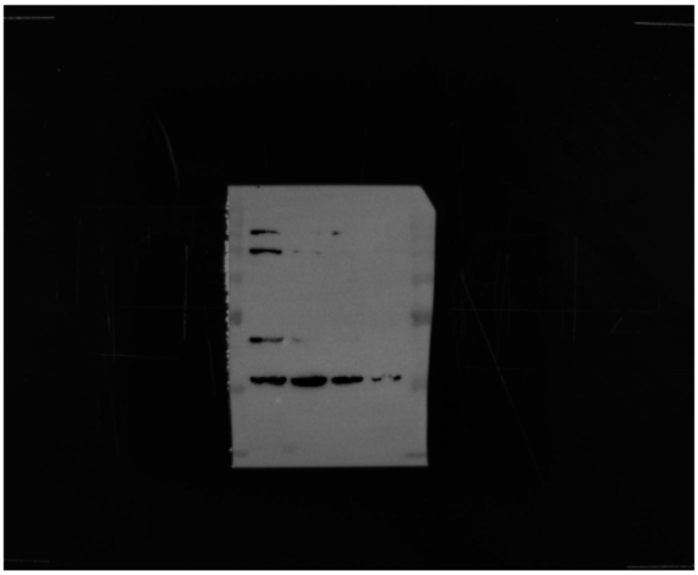

Figure 4 – Uncropped blot of STK38 (Related to Figure 4c)

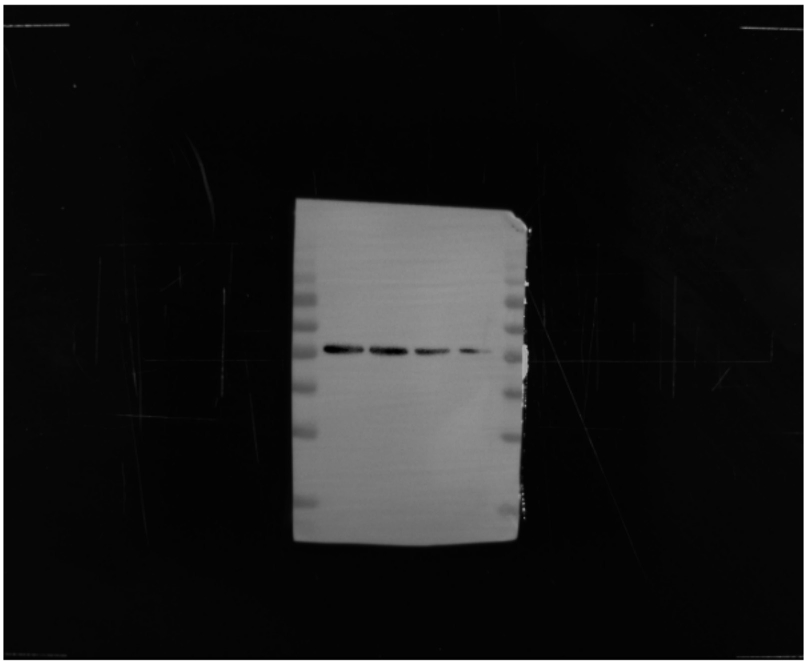

Figure 5 – Uncropped blot of caspase-3 (Related to Figure 5g)

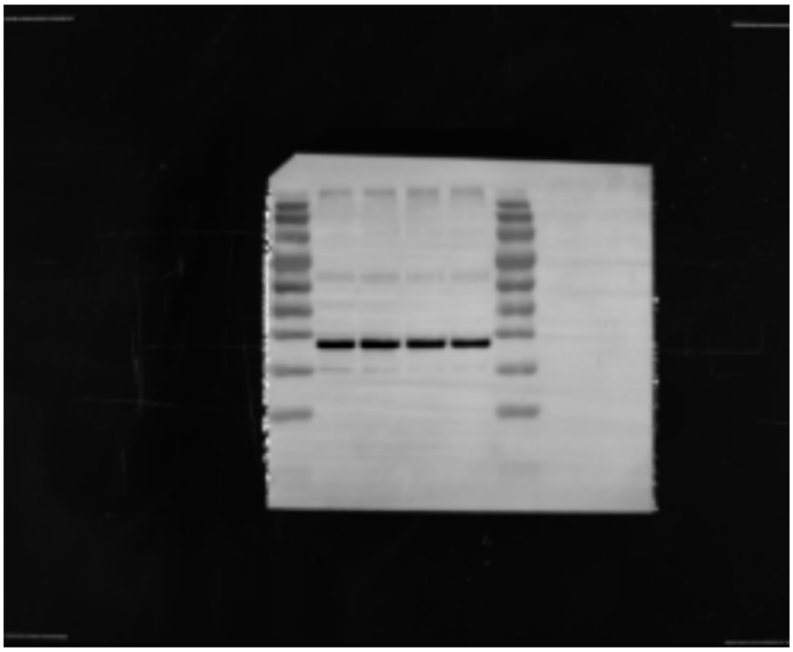

Figure 5 – Uncropped blot of Cit-H3 (Related to Figure 5g)

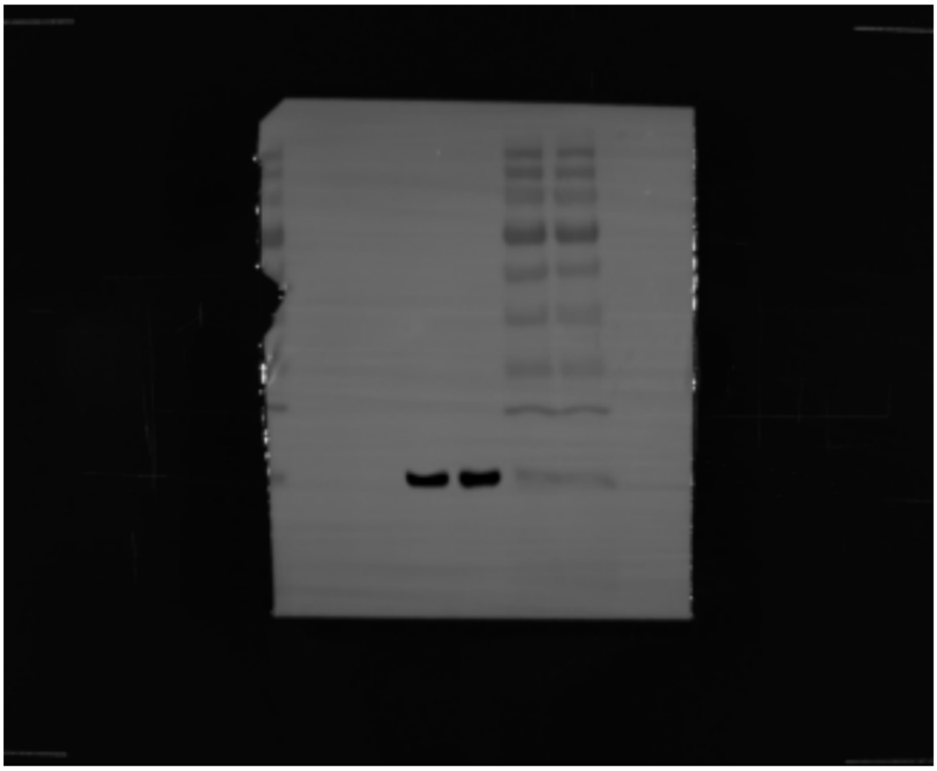

Figure 5 – Uncropped blot of GAPDH (Related to Figure 5g)

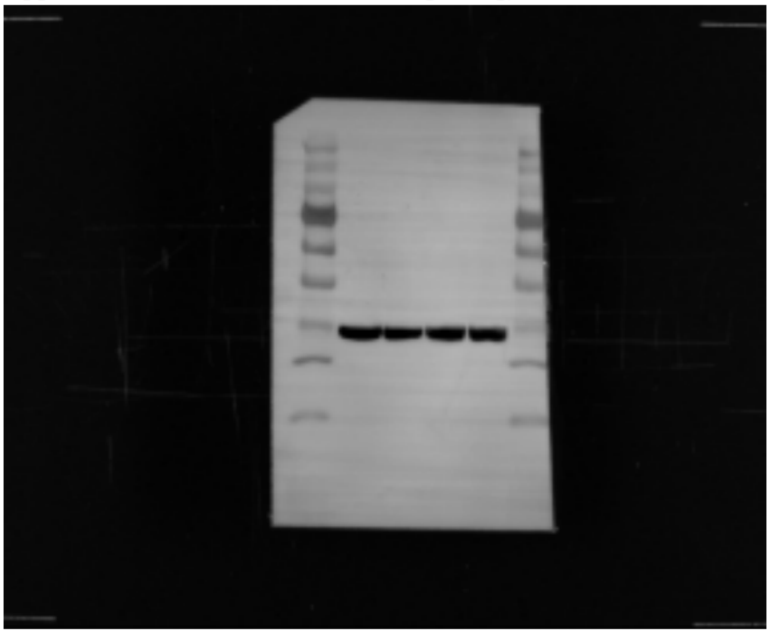

Figure 5 – Uncropped blot of Histone-H3 (Related to Figure 5g)

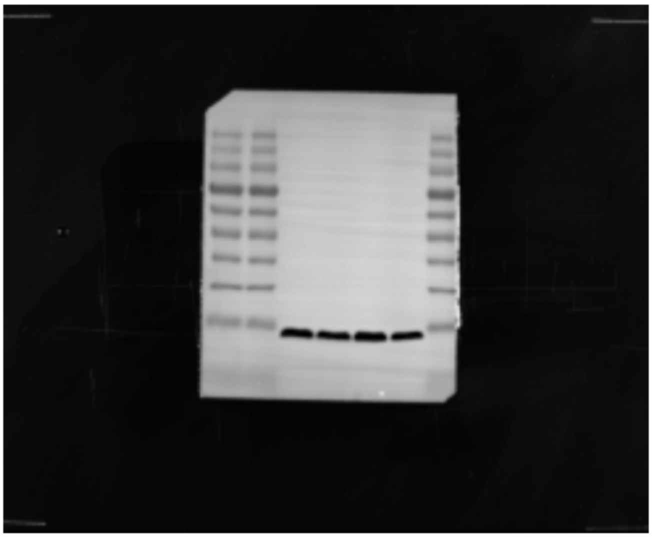

Figure 5 – Uncropped blot of LC3B (Related to Figure 5g)

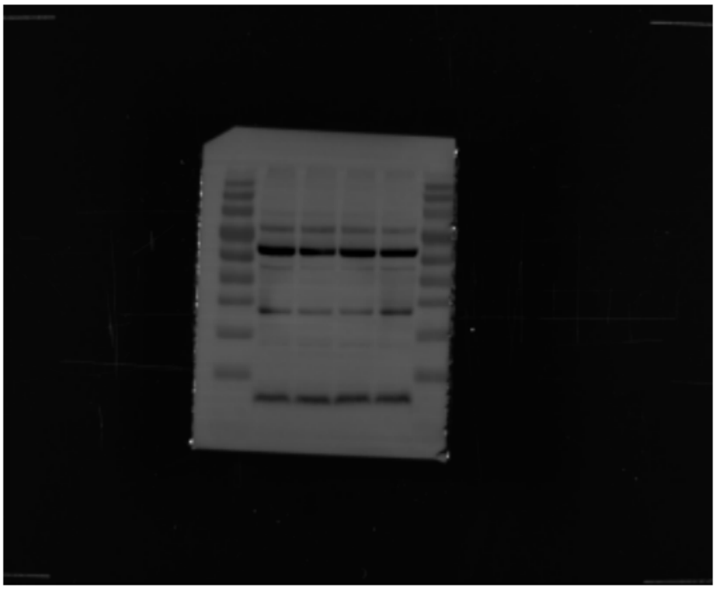

Figure 5 – Uncropped blot of STK38 (Related to Figure 5g)

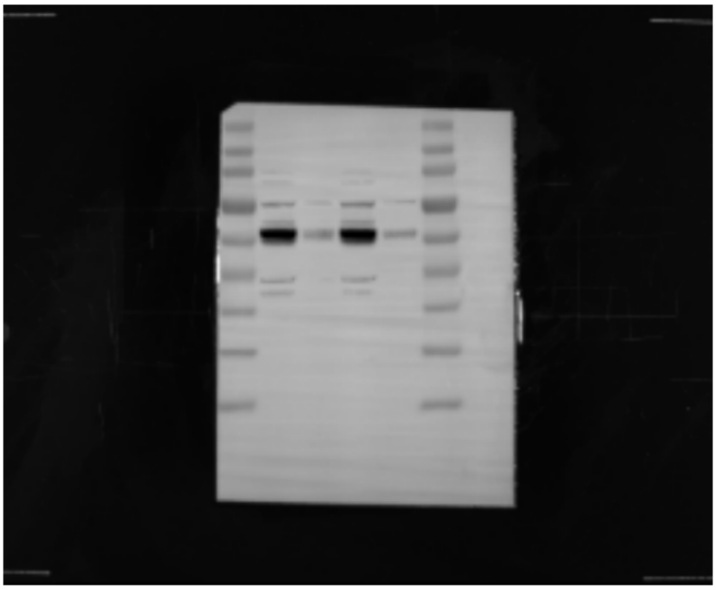

Figure S2 – Uncropped blot of GAPDH (Related to Figure S2 c)

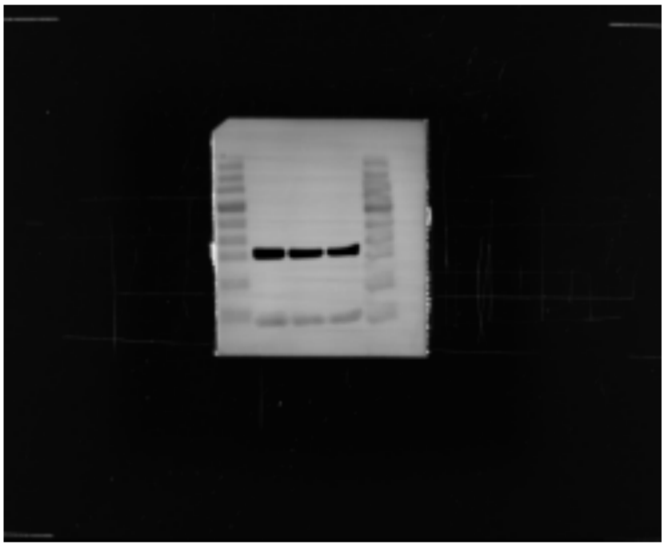

Figure S2 – Uncropped blot of KIF7 (Related to Figure S2 c)

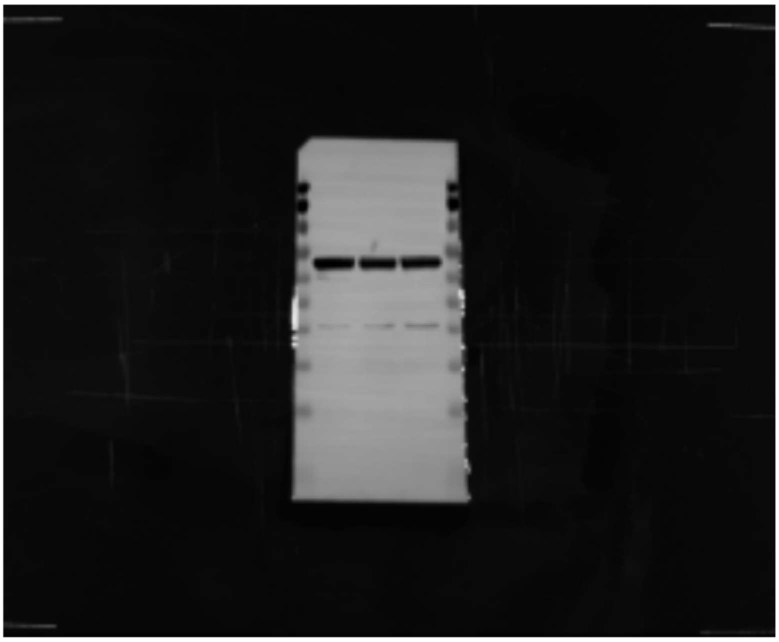

Figure S2 – Uncropped blot of KIF-Phostag (Related to Figure S2 c)

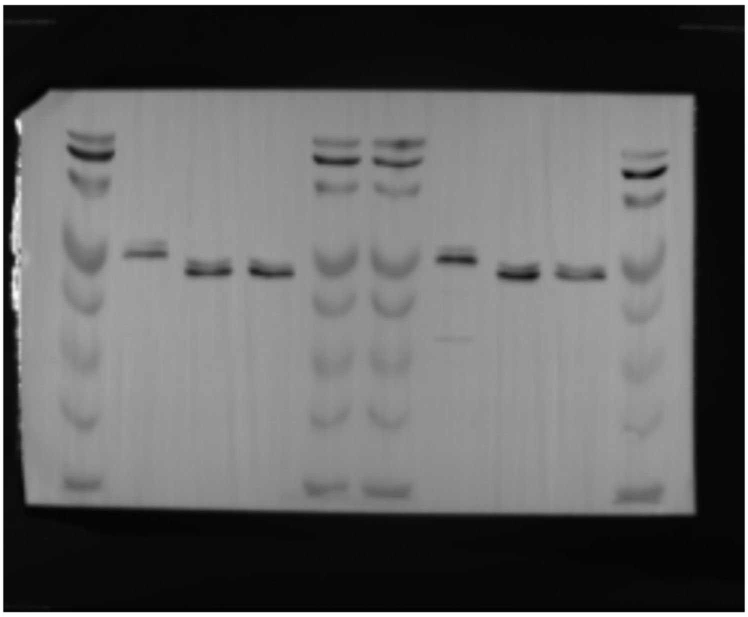

Figure S2 – Uncropped blot of STK38 (Related to Figure S2 c)

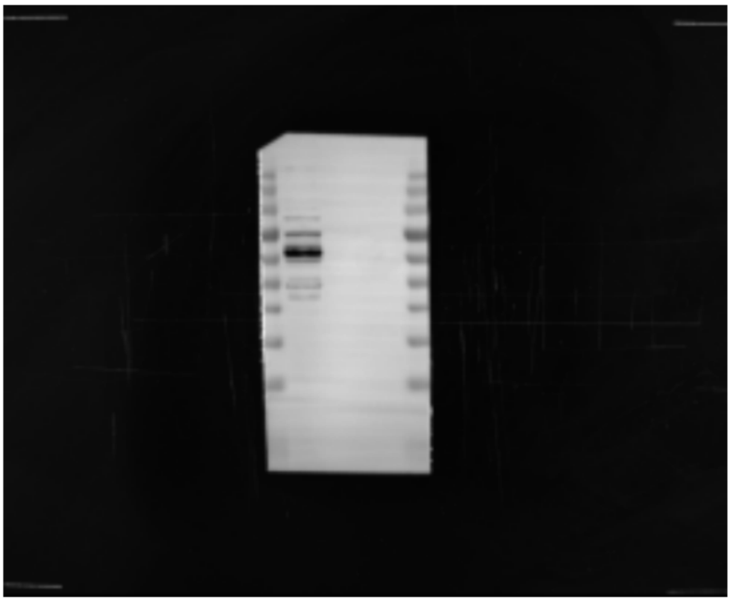

Figure S2 – Uncropped blot of ACTB (Related to Figure S2 d)

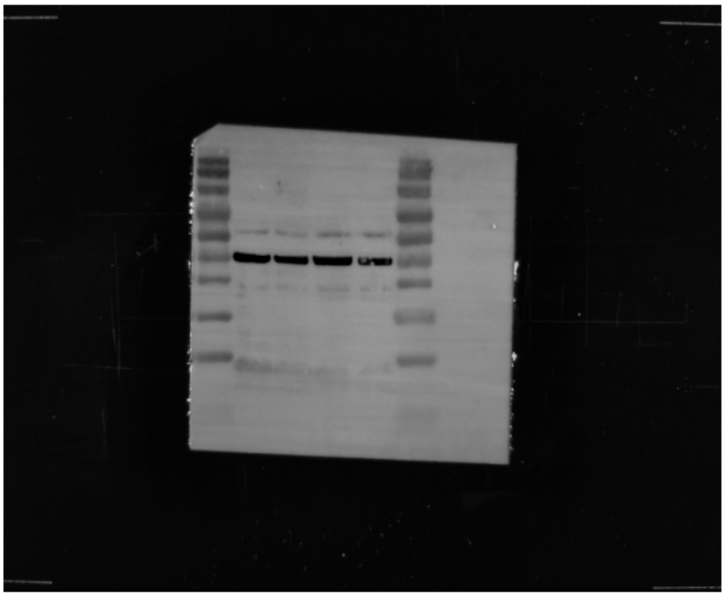

Figure S2 – Uncropped blot of GSK3 β (Related to Figure S2 d)

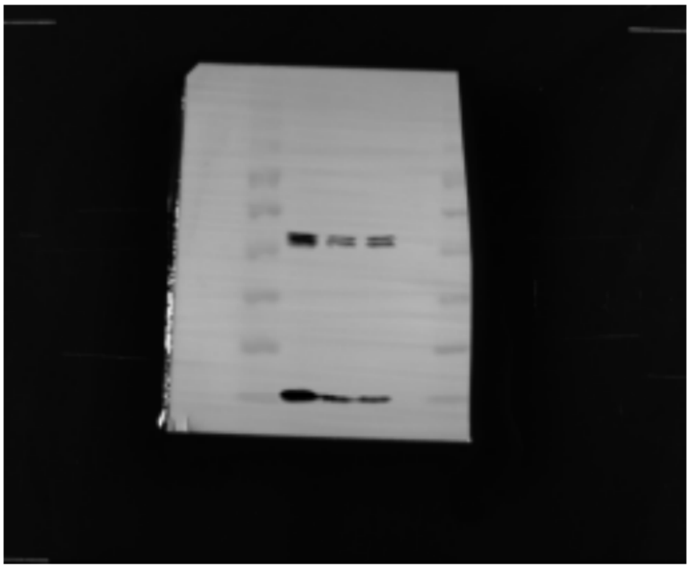

Figure S2 – Uncropped blot of ACTB (Related to Figure S2 e)

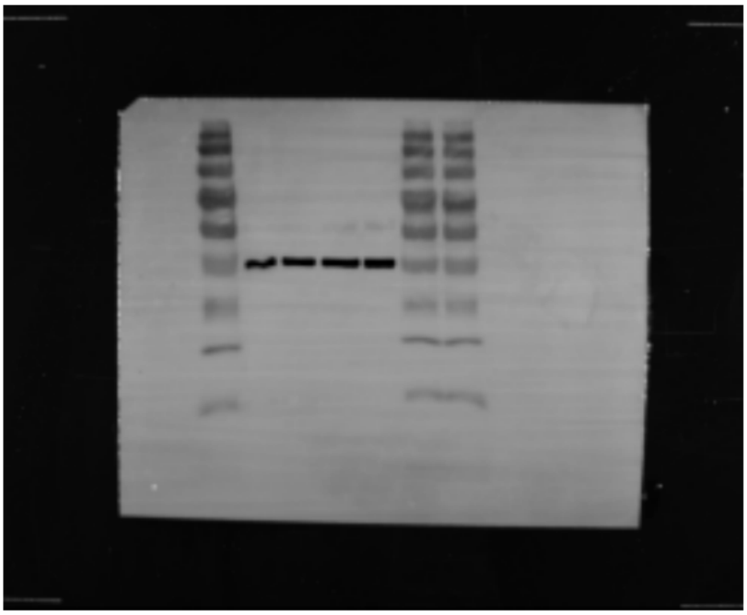

Figure S2 – Uncropped blot of KIF7 (Related to Figure S2 e)

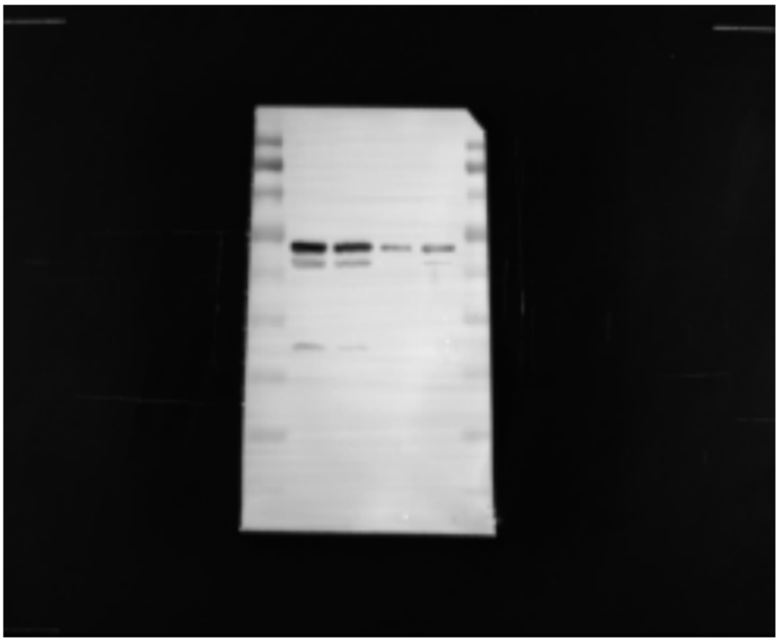

Supplement: Supplementary file 8 — Uncropped Western Blot Images [file 41419_2025_8225_MOESM8_ESM.pdf]
